# Active contact and follow-up interventions to prevent repeat suicide attempts during high-risk periods among patients admitted to emergency departments for suicidal behavior: a systematic review and meta-analysis

**DOI:** 10.1186/s12888-019-2017-7

**Published:** 2019-01-25

**Authors:** Masatoshi Inagaki, Yoshitaka Kawashima, Naohiro Yonemoto, Mitsuhiko Yamada

**Affiliations:** 1 Department of Psychiatry, Faculty of Medicine, Shimane University, 89-1, Enya-cho, Izumo-shi, Shimane 693-8501 Japan; 2 Department of Neuropsychopharmacology, National Institute of Mental Health, National Center of Neurology and Psychiatry, 4-1-1, Ogawahigashimachi, Kodaira, Tokyo, 187-8553 Japan; 3 Department of Biostatistics, Kyoto University School of Public Health, Yoshida Konoe-cho, Sakyo-ku, Kyoto, 606-8501 Japan

**Keywords:** Suicide, Self-harm, Emergency department, Meta-analysis, Systematic review

## Abstract

**Background:**

There is evidence that several intervention types, including psychotherapy, reduce repeat suicide attempts. However, these interventions are less applicable to the heterogeneous patients admitted to emergency departments (EDs). The risk of a repeat suicide attempt is especially high in the first 6 months after the initial attempt. Therefore, it is particularly important to develop effective ED interventions to prevent repeat suicide attempts during this 6-month period.

**Methods:**

We systematically reviewed randomized controlled trials of ED-initiated interventions for suicidal patients admitted to EDs using the databases MEDLINE, PsychoINFO, CINAHL, and EMBASE up to January 2015 in accordance with an a priori published protocol (PROSPERO: CRD42013005463). Interventions were categorized into four types, including active contact and follow-up interventions (intensive care plus outreach, brief interventions and contact, letter/postcard, telephone, and composite of letter/postcard and telephone), and a meta-analysis was conducted to determine pooled relative risks (RRs) and 95% confidence intervals (CIs) of a repeat suicide attempt within 6 months.

**Results:**

Of the 28 selected trials, 14 were active contact and follow-up interventions. Two of these trials (*n* = 984) reported results at 6 months (pooled RR = 0.48; 95% CI: 0.31 to 0.76). There were not enough trials of other interventions to perform meta-analysis. Some trials included in the meta-analysis were judged as showing risk of bias.

**Conclusion:**

Active contact and follow-up interventions are recommended for suicidal patients admitted to an ED to prevent repeat suicide attempts during the highest-risk period of 6 months.

**Systematic review registration:**

PROSPERO CRD42013005463 (27 August 2013).

**Electronic supplementary material:**

The online version of this article (10.1186/s12888-019-2017-7) contains supplementary material, which is available to authorized users.

## Background

Suicide is a critical international problem [1–3]. Prior suicide attempts and a history of self-harm behavior are the most predictive risks for death by suicide and suicide attempts [4, 5]. The risk of repeat suicide attempts is highest in the period immediately following a suicide attempt, and one in 10 patients repeat within 5 days (median first repetition: 83·5 days; interquartile range: 20 to 187 days) [6]. Therefore, it is important to develop effective interventions to prevent repeat suicide attempts during the highest-risk period of 6 months.

In England, 220,000 patients per year are admitted to the hospital for self-harm behaviors [7]. In the United States, 538,000 patients per year are admitted to the emergency departments (EDs) for attempted suicide and self-injury [8]. Therefore, ED is the one of the best settings in which effective interventions for such patients could be developed [9, 10].

There have been previous systematic reviews and meta-analyses of interventions for repeat suicide attempts, although these have not focused solely on ED settings. One previous systematic review showed that cognitive–behavioral therapy- based interventions for patients with a history of suicidal behaviors reduced repeated suicidal behaviors within 12 months [11]. Another systematic review of brief contact interventions (telephone, letter, or postcard) showed a reduction in the rate of repeated suicidal behaviors in 12 months [12]. One meta-analysis showed that psychosocial and behavioral interventions that directly address suicidal thoughts and behavior are effective post-treatment (mean duration: 11·3 months), whereas treatments that indirectly address these components are only effective long-term [13]. However, these studies did not report the results at 6 months and therefore have limited application to ED settings, although the risk of repeat suicide attempts is highest in the period immediately following a suicide attempt [6].

We previously performed a systematic review and meta-analysis of trials assessing the effects on repeated suicidal behavior of ED-initiated interventions for suicidal patients admitted to EDs [14]. In the previous review study [14], we categorized interventions by type. The categorization was carried out by the research team, which comprised psychiatrists and psychologists who had experience of working in suicide prevention at EDs. Intensive care plus outreach, brief intervention and contact, letter/postcard, telephone, and composite of letter/postcard and telephone were categorized as active contact and follow-up interventions. The active contact and follow-up interventions were developed empirically and were applicable to ED settings. The previous meta-analysis showed that, in nine trials, the interventions significantly reduced the risk of a repeat suicide attempt within 12 months [14]. Other types of intervention, including psychotherapy, had no significant effects on risk reduction [14]. However, the data did not indicate which interventions were effective in ED settings during the highest-risk period of 6 months, although research indicates that the risk of repeat suicide attempts is highest in the period immediately following a suicide attempt [6].

Since our previous systematic review and meta-analysis, the results of several trials evaluating the effect of interventions at 6 months have been published. Therefore, this study examines the effect of ED-initiated active contact and follow-up interventions on the risk of a repeat suicide attempt within 6 months in patients admitted to an ED for suicidal injury. We also examine the effect at 12 months as a secondary outcome.

## Methods

We conducted our systematic review and meta-analysis in accordance with the method used in our previous study [14] and an a priori published protocol (http://www.crd.york.ac.uk/PROSPERO/display_record.asp?ID=CRD42013005463), and have reported the results according to the PRISMA criteria for systematic reviews and meta-analyses [15]. Therefore, we briefly describe the method as follows.

### Search strategy

We conducted a search of the databases MEDLINE (from 1949), PsychINFO (from 1887), CINAHL (from 1981), and EMBASE (from 1974) from their inception to January 2015. Search terms were (suicide* OR self-harm* OR self harm* OR self-poison* OR overdose* OR self-injur*) AND (randomize* OR randomis*). We also examined the reference lists of identified studies for further references. We did not distinguish between suicide attempts and deliberate self-harm or self-injury in accordance with a previous report [16] and our previous study [14].

### Study eligibility

Inclusion criteria were as follows: all participants had attempted suicidal behavior within 1 month and had been admitted to an ED for their suicidal behavior, assessment for eligibility for initial interventions in the trial was performed while the patients were in the ED or a subsequent ward, and the effect of the intervention was examined using a randomized controlled trial and was described in the manuscript.

We determined the first two criteria in accordance with our previous systematic review and meta-analysis [14] to ensure that trial participants had been admitted to EDs and that interventions had been initiated during the ED admission. We focused on trials that included patients who had experienced serious injury as a result of their suicide behavior and who required ED admission, as such patients are likely to be at higher risk of repeat suicide [17]. It is probable that this criterion largely excluded patients displaying milder self-harm behaviors and included patients displaying severer suicidal behaviors with serious suicide intent [18].

### Exclusion criteria

The exclusion criteria were as follows: experimental interventions comprising only physical therapy for physical injury or poisoning, manuscripts not written in English, and studies in which the main outcome was a subgroup analysis of the trial.

### Data management

Summary tables were created by extracting data on type of intervention, number of participants, inclusion and exclusion criteria, adherence of participants to interventions, proportion of participants followed up for outcomes, and effects of the interventions on repeat suicidal behaviors and death by suicide. We extracted and summarized data on the psychological measures used as outcomes.

In accordance with our previous study [14], we classified the selected trials into four groups (active contact and follow-up interventions and the subtypes [e.g., intensive follow-up, outreach, case management, telephone call, and letter/post card interventions], psychotherapy [e.g., problem-solving approach, psychodynamic interpersonal therapy, cognitive/behavioral/cognitive–behavioral therapy], pharmacotherapy, and miscellaneous). The categories were determined by the researchers of the previous study, who were psychiatrists and psychologists with experience of working in suicide prevention at EDs.

### Assessment of bias

We assessed the risk of bias according to the Cochrane Handbook for Systematic Reviews of Interventions (Version 5.1.0) [19].

### Statistical analysis

We performed a meta-analysis to examine the effect of each type of intervention on a repeat suicide attempt during the 6 months. As a secondary analysis, we also performed a meta-analysis of the effect at 12 months to incorporate data that had been published since our previous meta-analysis [14].

We systematically reviewed all types of psychometric measure used in the selected trials. However, we could not analyze data from psychometric measures (such as measures of depression, hopelessness, and suicidal ideation) as outcomes. The reviewed trials used different kinds of psychometrics at the various measurement points. In addition, some trials used ad hoc questions that had not been validated.

The meta-analysis was performed using similar method to that in our previous meta-analysis [14] to determine pooled relative risks (RRs) and their 95% confidence intervals (CIs). A fixed-effects model using the Mantel–Haenszel method or a random-effects model using the DerSimonian–Laird method [20] was used.

## Results

**Fig. 1:**
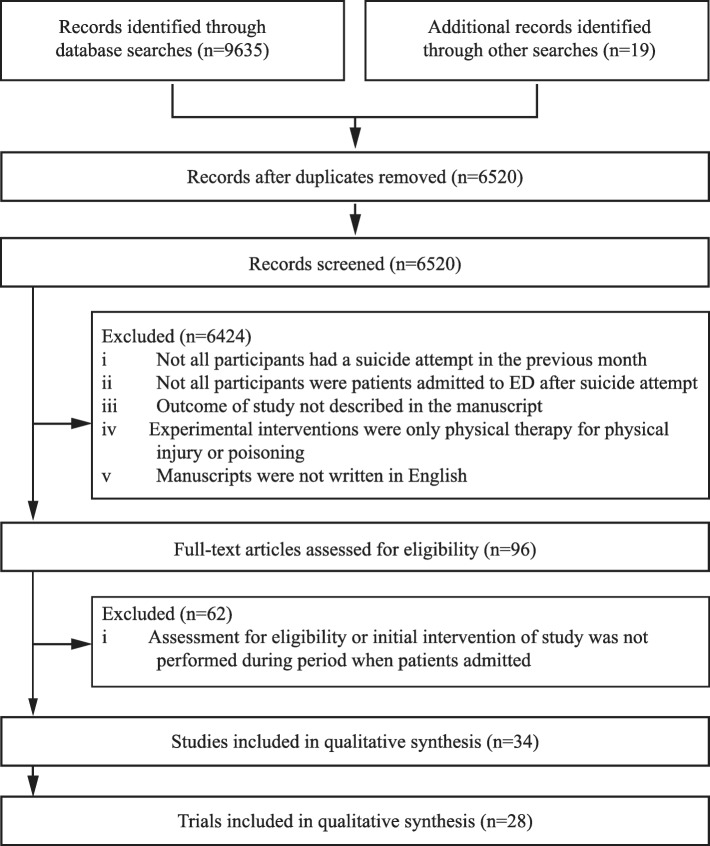
Study selection. Two and 11 trials, respectively, were included in a meta-analysis of the effect of active contact and follow-up interventions on repeat suicide attempts at 6 and 12 months. There have been no new publications on psychotherapy and pharmacotherapy interventions for suicide attempts since our previous meta-analysis. Therefore, we did not perform meta-analyses on the effect of these interventions in the present study

From 9654 records identified through database searches and other searches, 6520 articles were retrieved after duplicates were removed. Of the 6520 articles, we included 28 trials that reported results in 34 publications [21–54] of any interventions initiated at an ED for admitted suicidal patients (Additional file 1: Table S1: List of selected trials and publications, Additional file 2: References in the additional files, and Fig. 1).

We classified the 28 trials (Additional file 1: Table S1) into four categories by intervention type: active contact and follow-up interventions (Table 1) (18 publications from 14 trials) [21, 24, 25, 27–30, 33, 37–41, 44, 45, 50–52], psychotherapy (12 publications from 10 trials) [22, 26, 32, 34–36, 42, 43, 46–48, 54], pharmacotherapy (1 publication from 1 trial) [23], and miscellaneous interventions (3 publications from 3 trials) [31, 49, 53]. Fourteen trials in 18 publications were active contact and follow-up interventions (Table 1). Ten trials were in the psychotherapy group, one in the pharmacotherapy group, and three in the miscellaneous group. We have listed the publications on psychotherapy, pharmacotherapy, and miscellaneous interventions and described the contents of the interventions in each trial in Additional file 3: Table S2. We have summarized the results (e.g., number of patients making suicide re-attempts, suicidal deaths, and any-cause deaths) of each publication in Additional file 4: Table S3.

**Table 1:** Active contact and follow-up interventions

	Intervention 1	Intervention 2/Comparison intervention	Control (TAU, Placebo)
Intensive care plus outreach			
Allard et al. 1992 [21]	Intensive follow-up with scheduled visits	–	TAU: care by regular hospital personnel
Van Heeringen et al. 1995 [52]	Home visit by nurse to patients who did not keep outpatient appointment	–	TAU: outpatient appointment
van der Sande et al. 1997 [51]	Intensive inpatient and community intervention	–	TAU: routine clinical service
Morthorst et al. 2012 [44]	Assertive intervention with outreach consultations	–	TAU: referral to a range of different treatment modalities
Kawanishi et al. 2014 [41]^a^	Assertive and continuous case management	–	TAU: enhanced usual care
Hatcher et al. 2015 [39]^a^	Support for up to 2 wk. and 4–6 sessions problem-solving therapy in 4 wk. followed by 8 postcards	–	TAU: referrals to multidisciplinary teams, crisis teams, and/or recommendations for engagement with community alcohol and drug treatment centers
Brief intervention and contact			
Fleischmann et al. 2008 [33]; Bertolote et al. 2010 [25]	Brief intervention and contact	–	TAU: the norms prevailing in the respective emergency departments
Mousavi et al. 2014 [45]^a^	Brief interventional contact followed by 7 follow-up telephone contacts	–	Brief interventional contact followed by treatment as usual
Letter or postcard			
Carter et al. 2005 [27], 2007 [28], 2013 [29]	Postcard sent	–	TAU: assessment and diagnosis by a psychiatrist
Beautrais et al. 2010 [24]	Postcard sent	–	TAU: assessment and referral to community-based mental health services
Hassanian-Moghaddam et al. 2011 [37], 2015 [38]^a^	Postcard sent	–	TAU: follow-up care was not coordinated
Telephone			
Cedereke et al. 2002 [30]	Telephone call at 4 and 8 mo	–	TAU: assessment by a psychiatrist and a social counsellor and referral to further general psychiatry treatment
Vaiva et al. 2006 [50]	Telephone call from psychiatrists at 1 mo	Telephone call from psychiatrists at 3 mo	TAU: no telephone contact
Composite of letter/postcard and telephone			
Kapur et al. 2013 [40]	Information leaflet, two telephone calls within the first 2 wk., and a series of 6 letters over a 12-mo period	–	TAU: a mental health liaison nursing team to carry out specialist assessments

We referred to and modified data from a previous paper by Inagaki et al. (2015), and we reviewed newly published studies^a^ and added new data to the present table

Abbreviations: *wk* week/weeks, *mo* month/months, *TAU* treatment as usual

The characteristics of the included studies are shown in Additional file 5: Table S4: Subjects, Additional file 6: Table S5: Adherence to intervention and follow-up rate, and Additional file 7: Table S6: Measures of suicidal behaviors. The number of trial participants varied from 18 [22] to 2300 [37, 38]. The psychotherapy group contained a relatively small number of participants (from 18 [22] to 400 [35]) compared with the active contact and follow-up group (from 66 [40] to 2300 [37, 38]). As shown in Additional file 6: Table S5, intervention adherence and follow-up rate were not high, suggesting possible bias in the trials.

The results of the psychometric measurements and other outcome measures used in the selected trials are shown in Additional file 8: Table S7a–7 h and Additional file 9: Table S8, respectively. A considerable variety of psychometric measures were used, including ad hoc questions. Not all psychometric measures had been validated or had associated reliability data. Trials measured not only suicidal ideation but also hopelessness, sense of belonging, depression, anxiety, general mental health, alcohol-related problems, quality of life, global functioning, problem solving, and other factors. Among the psychometric measures, the Beck Hopelessness Scale (BHS), the Scale for Suicide Ideation (SSI), and the Beck Depression Inventory (BDI) were validated, and the data were used to predict suicidal behavior and/or suicidal ideation (Additional file 8: Table S7). The BHS was used in seven trials (reported in eight publications), the SSI was used in seven trials (eight publications), and the BDI was used in five trials (six publications), making these the main psychometric measures used in the included trials.

Additional file 10: Table S9 shows the results of the risk of bias assessment. Many trials showed a high risk of bias, and most trials did not include information about blinding of participants and personnel.

**Fig. 2:**
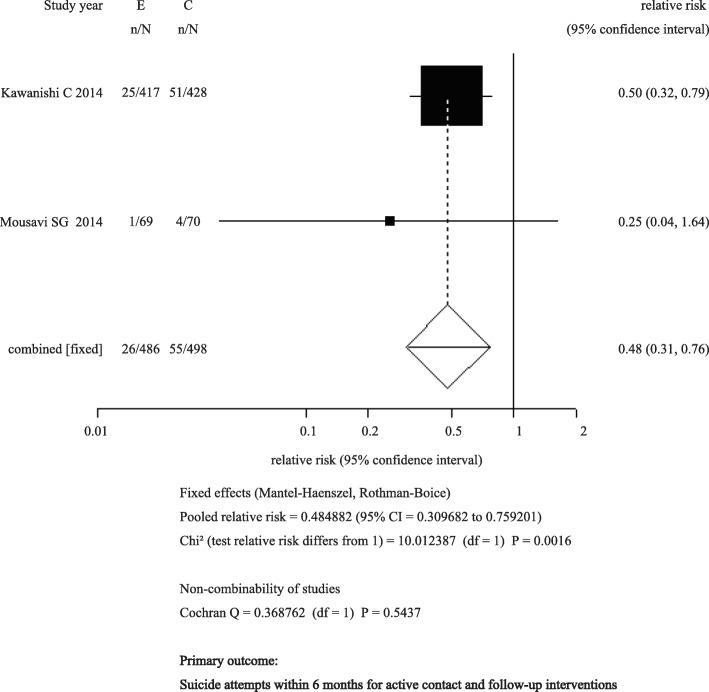
Primary outcome: Suicide attempts within 6 months for active contact and follow-up interventions. Two trials of active contact and follow-up interventions reported suicide attempts within 6 months [41, 45]. Two trials were included in the meta-analysis [41, 45]. The number of included participants and the number of participants who made repeat suicide attempts in each trial are shown in Table 1. To assess heterogeneity, we used the Cochrane Q statistic to examine heterogeneity among the trials in each analysis. We regarded heterogeneity as substantial if the Cochrane Q test produced a low *p*-value (< 0.10)

We extracted the intervention results for selected trials by suicide behavior (repeat suicide attempt, and suicidal death) and any cause of death. Active contact and follow-up intervention results are shown in Table 2, and results for other types of intervention are shown in Additional file 4: Table S3. We performed a meta-analysis of the effect of the active contact and follow-up interventions on repeat suicide attempts at 6 months as a primary meta-analysis and at 12 months as a secondary meta-analysis. The results of the primary meta-analysis examining the effects at 6 months are shown in Fig. 2. As the results of the systematic review, this meta-analysis included two trials [41, 45] (*n* = 984). There was a statistically significant effect of the intervention on prevention of a repeat suicide attempt (RR: 0.48; 95% CI: 0.31–0.76). The results suggest that active contact and follow-up interventions reduce the risk of a repeat suicide attempt within 6 months in patients admitted to an ED with suicidal injury. The meta-analysis included trials by Kawanishi et al. [41] and Mousavi et al. [45]. The intervention in the trial by Kawanishi et al. was called ACTION-J. It comprised assertive case management (based on psychiatric diagnoses, social risks, and patient needs) that included periodic contact with participants during their ED stay and after discharge, encouragement of participants to adhere to psychiatric treatment, coordination of appointments with psychiatrists and primary care physicians, referrals to social services and private support organizations, coordination of the use of these resources to accommodate the individual needs of patients, and provision of psychoeducational content and information about social resources [41]. The intervention in the trial by Mousavi et al. constituted seven follow-up telephone contacts after discharge in the second and fourth weeks, and in the second, third, fourth, fifth, and sixth months, by a final-year psychiatric resident [45].

**Table 2:** Results of active contact and follow-up interventions

	Re-attempts		Death	
	No. of patients with re-attempts/No. of patients in each group analysis	No. of re-attempts/No. of patients in each group analysis	No. of any-cause deaths/No. of patients in each group analysis	No. of suicidal deaths/No. of patients in each group analysis
Intensive care plus outreach				
Allard et al. 1992 [21]	• E: 22/63; C: 19/63	• E: 60/63; C: 54/63	–	• E: 3/76; C 1/74
Van Heeringen et al. 1995 [52]	• E: 21/196; C: 34/195	–	• 15 died in both groups	• E: 6/196; C: 7/195
van der Sande et al. 1997 [51]	• E: 24/140; C: 20/134	• E: 32/140; C: 31/134	–	• E: 1/140; C: 2/134
Morthorst et al. 2012 [44]	• E: 20/123; C: 13/120 (medically recorded) • E: 11/95; C: 13/74 (self-reported)	–	• E: 2/123; C: 1/120	• E: 1/123; C: 0/120
Kawanishi et al. 2014 [41]^a^	• E: 3/444; C: 16/445 in 1 mo • E: 7/430; C: 32/440 in 3 mo • E: 25/417; C: 51/428 in 6 mo • E: 43/397; C: 60/399 in 12 mo • E: 55/380; C: 71/385 in 18 mo	–	• E: 46/460; C: 42/454 during the overall study period	• E: 27/460; C: 30/454 during the overall study period
Hatcher et al. 2015 [39]^a^	• E: 47/327; C: 42/357 in 3 mo • E: 66/327; C: 73/357 in 12 mo • E: 86/737; C: 75/737 in 3 mo • E: 142/737; C: 135/737 in 12 mo	• E: 60/327; C: 62/357 in 3 mo • E: 129/327; C: 163/357 in 12 mo • E: 114/737; C: 108/737 in 3 mo • E: 256/737; C: 272/737 in 12 mo	• E: 2/327; C: 4/357 in 12 mo • E: 2/737; C: 4/737 in 12 mo	–
Brief intervention and contact				
Fleischmann et al. 2008 [33]; Bertolote et al. 2010 [25]	• E: 66/863; C: 60/800	–	• E: 11/872; C: 22/827	• E: 2/872; C: 18/827
Mousavi et al., 2014 [45]^a^	• E: 1/69; C: 4/70 in 6 mo	–	–	–
Letter or postcard				
Carter et al. 2005 [27], 2007 [28], 2013 [29]	• E: 57/378; C: 68/394 in 12 mo • E: 80/378; C: 90/394 in 24 mo • E: 94/378; C: 107/394 in 60 mo	• E: 101/378; C: 192/394 in 12 mo • E: 145/378; C: 310/394 in 24 mo • E: 252/378; C: 484/394 in 60 mo	• E: 22/378; C: 22/394 in 60 mo	• E: 5/378; C: 6/394 in 60 mo
Beautrais et al. 2010 [24]	• E: 39/153; C: 49/174	• E: 87/153; C: 136/174	–	–
Hassanian-Moghaddam et al. 2011 [37], 2015 [38]^a^	• E: 31/1043; C: 55/1070 in 12 mo • E: 62/997; C: 91/1004 in 24 mo	• E: 34/1043; C: 58/1070 in 12 mo	• E: 7/1150; C: 2/1150 in 12 mo • E: 8/1150; C: 5/1150 in 24 mo	• E: 8/1150; C: 4/1150 in 24 mo
Telephone				
Cedereke et al. 2002 [30]	• E: 14/83 vs. C: 15/89	• E: 26/83 vs. C: 27/89	–	• E: 1/107; C: 1/109
Vaiva et al. 2006 [50]	• E1: 24/147; E2: 20/146; C: 59/312	–	• 6 died in three groups	• E1: 0/147; E2: 1/146; C: 2/312
Composite of letter/postcard and telephone				
Kapur et al. 2013 [40]	• E: 11/33; C: 4/32	• E: 41/33; C: 7/32	• E: 1/33; C:0/32	–

We referred to and modified data from a previous paper by Inagaki et al. (2015), and we reviewed newly published studies^a^ and added new data to the present table

Abbreviations: *E* experimental intervention group, *C* control group

None of the nine selected trials of psychotherapy interventions examined the effect on a repeat suicide attempt at 6 months. There was only one trial of a pharmacotherapy intervention, which did not report the effects on a repeat suicide attempt at 6 months.

**Fig. 3:**
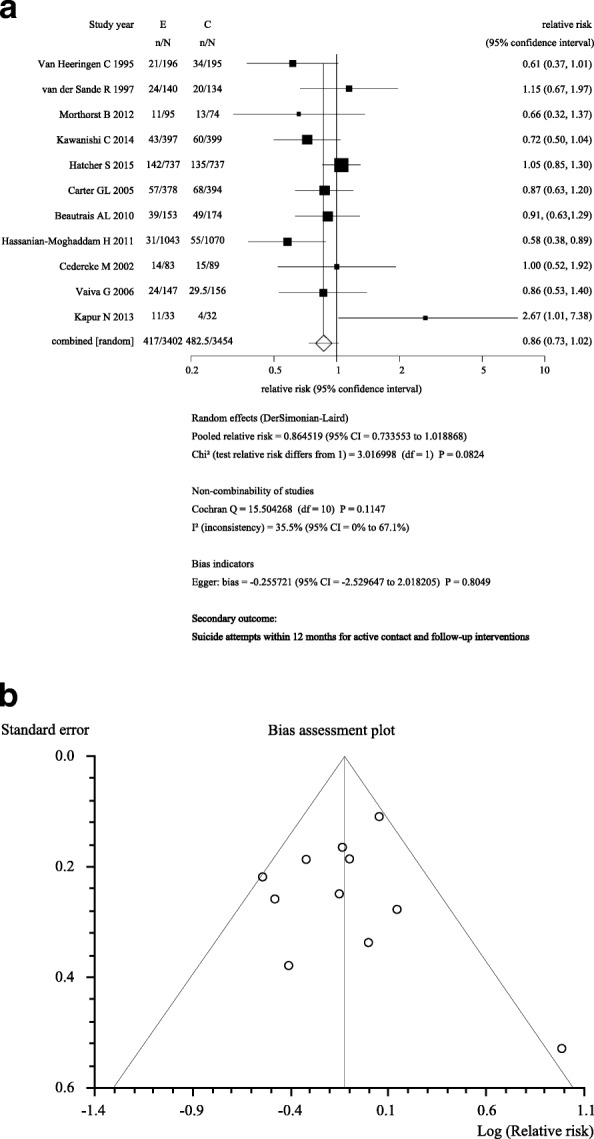
Secondary Outcome: Suicide attempts within 12 months for active contact and follow-up interventions. **a** The meta-analysis included 11 trials [24, 27, 30, 37, 39–41, 44, 50–52]. The number of included participants and the number of participants who made repeat suicide attempts in each trial are shown in Table 1. To assess heterogeneity, we used the I^2^ and Cochrane Q statistics to examine heterogeneity among the trials in each analysis. We regarded heterogeneity as substantial if I^2^ was greater than 30% or if the Cochrane Q test produced a low *p*-value (< 0.10). **b** We investigated publication bias by constructing a funnel plot and by using the Egger test

As our secondary meta-analysis in addition to the primary meta-analysis of the effect at 6 months, we also examined the effect of active contact and follow-up interventions at 12 months to incorporate data that had been published since our previous meta-analysis [14]. Figure 3a and b shows the results of the meta-analysis of 11 trials (*n* = 6859) for a repeat suicide attempt within 12 months. Two new trials [39, 41] were added to our previous meta-analysis [14]. The risk of a repeat suicide attempt was reduced, but this was not statistically significant (RR: 0.86; 95% CI: 0.73–1.02). Among the trials included in the meta-analysis, the study by Hatcher et al. [39] used the Zelen design. Although 737 patients were randomly allocated to the intervention group, only 327 participants consented to receive the intervention. Of the remaining 737 patients randomly allocated to the treatment as usual group, only 357 consented to receive treatment-as-usual and to be followed up. The intent-to-treat (ITT) analysis included those patients who did not consent to receive the interventions (*n* = 410 in the intervention group and *n* = 380 in the treatment-as-usual group). This may have diluted the effect of the interventions. To avoid this problem, we performed a post hoc meta-analysis excluding the Hatcher et al. trial and found a significant reduction in the risk of a repeat suicide attempt at 12 months (RR: 0.82; 95% CI: 0.69–0.98).

Hatcher et al. also performed per-protocol-based (PPB) analysis using a different analysis set comprising participants who consented to receive the intervention (*n* = 327) and treatment as usual (*n* = 357). Another study by Morhorst et al. [44] reported results from two types of outcome measure: medical records and patient self-reports. We suspected that a meta-analysis using combinations of the two analysis sets from the Hatcher et al. trial and the two different outcomes from the Morhorst et al. trial [44] would show different results. The meta-analysis results for the four patterns [2 (ITT and PPB) by 2 (medical records and self-reports)] are shown in Additional file 11: Table S10: Four patterns of suicide attempts within 12 months in the active contact and follow-up group.

There have been no newly published trials of psychotherapy or pharmacotherapy interventions for suicide attempts within 6 months and 12 months since our previous meta-analysis [14].

## Discussion

The present study focused on the prevention of repeat suicide attempts during the highest-risk period after a suicide attempt (within 6 months). Our meta-analysis showed that active contact and follow-up interventions were effective in preventing a repeat suicide attempt within 6 months in patients admitted to EDs for suicidal injury.

The selected trials used ED-initiated interventions. Thus, compared with findings from interventions developed for other settings, the present findings are more applicable to high-risk patients admitted to EDs for suicidal behavior. Previous meta-analyses of cognitive–behavioral therapy-based interventions [11] and brief contact interventions [12] are not specific to patients admitted to EDs.

Active contact and follow-up interventions may reduce the risk of a repeat suicide attempt within 12 months. In the present meta-analysis, two trials (publications No. 5 by Kawanishi et al. and No. 6 by Hatcher et al. in Additional file 1: Table S1) [39, 41] were added to nine trials included in our previous meta-analysis [14]. One of the trials by Hatcher et al. used the Zelen design. Of the 737 patients randomly allocated to the intervention group, more than half (410 patients: 56%) did not consent to receive the intervention. This may have diluted the effect analyzed in the present meta-analysis. The post hoc meta-analysis excluding this report showed a significant reduction of the risk at 12 months.

The present findings demonstrated that active contact and follow-up type interventions were effective in reducing the risk of a repeat suicide attempt within 6 months. Active contact and follow-up interventions could reinforce connectedness among patients and care providers. However, the precise mechanisms by which the interventions reduce repeat suicide attempts are unclear, and further research is needed.

As previously proposed [55], it is very important to provide care to adolescents and young adults who self-harm and are admitted to EDs. Fifty-eight percent of participants in the Mousavi et al. trial (included in the present meta-analysis of suicide attempts within 6 months) were aged between 15 and 25 years. However, the Kawanishi et al. trial excluded patients younger than 20 years. Therefore, the present findings regarding the effect of active contact and follow-up interventions may not be generalizable to a young population.

The present findings are not conclusive regarding the effect at 6 months of ED-initiated psychotherapy interventions to reduce the risk of a repeat suicide attempt among patients admitted to an ED for suicidal injury. There were too few trials of psychotherapy interventions to perform a meta-analysis of the effect on a repeat suicide attempt at 6 months. More trials with large samples measuring the effect of interventions on suicide attempts are needed.

Some of the selected trials did not report figures for suicide attempts (Table 2). The number of suicide attempts was small, even in trials reporting the outcome. Most of the selected trials used various psychometric measures (Additional file 8: Table S7). The BHS, SSI, and BDI were the most frequently used measures, and have been previously validated and shown to predict suicide behavior. The use of such validated and standardized psychometric measures as a core outcome set is recommended and could facilitate future meta-analysis of the effect of interventions.

This study has several limitations. First, some of the interventions included may have beneficial effects on other psychological symptoms, and not all interventions reduced repeat suicide attempts. Second, although the trials included in the meta-analysis used control groups receiving treatment as usual, these treatments probably differed across studies. Third, for convenience, we categorized interventions into an active contact and follow-up group; however, the interventions within this group may have been different. Finally, some trials included in the meta-analysis were judged as showing risk of bias (Additional file 10: Table S9).

## Conclusions

In summary, the meta-analysis results indicate that active contact and follow-up interventions reduce the risk of a repeat suicide attempt within 6 months in patients admitted to an ED with suicidal injury. We recommend that this type of intervention be implemented to reduce patients’ suicide attempts. The findings may have implications for future clinical policy-making on the prevention of repeat suicidal behavior. This type of intervention could be adopted throughout EDs to reduce the risk of repeat suicide attempts.

## Additional files

Additional file 1: List of selected publications. (DOCX 34 kb)

Additional file 2: References in the additional files. (DOCX 27 kb)

Additional file 3: Interventions (psychotherapy, pharmacotherapy, and miscellaneous interventions). (DOCX 30 kb)

Additional file 4: Results (psychotherapy, pharmacotherapy, and miscellaneous interventions). (DOCX 34 kb)

Additional file 5: Subjects. (DOCX 84 kb)

Additional file 6: Adherence to intervention and follow-up rate. (DOCX 84 kb)

Additional file 7: Measure of suicidal behaviors. (DOCX 87 kb)

Additional file 8: List of psychometric measures. a: Suicidal ideation. b: Hopelessness. c: Sense of belonging. d: Depression, anxiety, and general mental health. e: Alcohol-related problems. f: Quality of life and global functioning. g: Problem solving. h: Others. (DOCX 359 kb)

Additional file 9: List of other outcomes. (DOCX 244 kb)

Additional file 10: Risk of bias. (DOCX 82 kb)

Additional file 11: Four patterns of suicide attempts within 12 months in the active contact and follow-up group. (DOCX 33 kb)

## Abbreviations

BDI: Beck depression inventory
BHS: Beck hopelessness scale
CI: Confidence interval
ED: Emergency departments
ITT: Intent-to-treat
PPB: Per-protocol-based
RR: Relative risk
SSI: Scale for suicide ideation
