# Supplementary material for: Active contact and follow-up interventions to prevent repeat suicide attempts during high-risk periods among patients admitted to emergency departments for suicidal behavior: a systematic review and meta-analysis
Source: BMC Psychiatry. 2019 Jan 25;19:44. doi: 10.1186/s12888-019-2017-7 (PMC6347824; doi:10.1186/s12888-019-2017-7)
Supplement: Supplementary file 1 — List of selected publications. (DOCX 34 kb) [file 12888_2019_2017_MOESM1_ESM.docx]

**Table S1 List of selected trials and publications**

| **Trial No.** | **Reference No. (see Additional file 11). Authors. Title. Journal year; volume(issue): pages** |
| --- | --- |
| **Active contact and follow-up group (Intensive care plus outreach)** | |
| 1 | 2. Allard R, Marshall M, Plante MC. Intensive follow-up does not decrease the risk of repeat suicide attempts. *Suicide Life Threat Behav* 1992; **22**(3): 303–14. |
| 2 | 3. Van Heeringen C, Jannes S, Buylaert W, Henderick H, De Bacquer D, Van Remoortel J. The management of non-compliance with referral to out-patient after-care among attempted suicide patients: a controlled intervention study. *Psychol Med* 1995; **25**(5): 963–70. |
| 3 | 4. van der Sande R, van Rooijen L, Buskens E, et al. Intensive in-patient and community intervention versus routine care after attempted suicide. A randomised controlled intervention study. *Br J Psychiatry* 1997; **171**: 35–41. |
| 4 | 5. Morthorst B, Krogh J, Erlangsen A, Alberdi F, Nordentoft M. Effect of assertive outreach after suicide attempt in the AID (assertive intervention for deliberate self harm) trial: randomised controlled trial. *BMJ* 2012; **345**: e4972. |
| 5 | 6. Kawanishi C, Aruga T, Ishizuka N, et al. Assertive case management versus enhanced usual care for people with mental health problems who had attempted suicide and were admitted to hospital emergency departments in Japan (ACTION-J): a multicentre, randomised controlled trial. *Lancet Psychiatry* 2014; **1**(3): 193–201. |
| 6 | 7. Hatcher S, Sharon C, House A, Collins N, Collings S, Pillai A. The ACCESS study: Zelen randomised controlled trial of a package of care for people presenting to hospital after self-harm. *Br J Psychiatry* 2015; **206**(3): 229–36. |
| **Active contact and follow-up group (Brief intervention and contact)** | |
| 7 | 8. Fleischmann A, Bertolote JM, Wasserman D, et al. Effectiveness of brief intervention and contact for suicide attempters: a randomized controlled trial in five countries. *Bull World Health Organ* 2008; **86**(9): 703–9.  9. Bertolote JM, Fleischmann A, De Leo D, et al. Repetition of suicide attempts: data from emergency care settings in five culturally different low- and middle-income countries participating in the WHO SUPRE-MISS Study. *Crisis* 2010; **31**(4): 194–201. |
| 8 | 10. Mousavi SG, Zohreh R, Maracy MR, Ebrahimi A, Sharbafchi MR. The efficacy of telephonic follow-up in prevention of suicidal reattempt in patients with suicide attempt history. *Adv Biomed Res* 2014; **3**: 198. |
| **Active contact and follow-up group (Letter or postcard)** | |
| 9 | 11. Carter GL, Clover K, Whyte IM, Dawson AH, D'Este C. Postcards from the EDge project: randomised controlled trial of an intervention using postcards to reduce repetition of hospital treated deliberate self poisoning. *BMJ* 2005; **331**(7520): 805.  12. Carter GL, Clover K, Whyte IM, Dawson AH, D'Este C. Postcards from the EDge: 24-month outcomes of a randomised controlled trial for hospital-treated self-poisoning. *Br J Psychiatry* 2007; **191**: 548–53.  13. Carter GL, Clover K, Whyte IM, Dawson AH, D'Este C. Postcards from the EDge: 5-year outcomes of a randomised controlled trial for hospital-treated self-poisoning. *Br J Psychiatry* 2013; **202**(5): 372–80. |

**Table S1 List of selected trials and publications (continued)**

| **Trial No.** | **Reference No. (see Additional file 11). Authors. Title. Journal year; volume(issue): pages** |
| --- | --- |
| **Active contact and follow-up group (Letter or postcard) (continued)** | |
| 10 | 14. Beautrais AL, Gibb SJ, Faulkner A, Fergusson DM, Mulder RT. Postcard intervention for repeat self-harm: randomised controlled trial. *Br J Psychiatry* 2010; **197**(1): 55–60. |
| 11 | 15. Hassanian-Moghaddam H, Sarjami S, Kolahi AA, Carter GL. Postcards in Persia: randomised controlled trial to reduce suicidal behaviours 12 months after hospital-treated self-poisoning. *Br J Psychiatry* 2011; **198**(4): 309–16.  16. Hassanian-Moghaddam H, Sarjami S, Kolahi AA, Lewin T, Carter G. Postcards in Persia: A twelve to twenty-four month follow-up of a randomized controlled trial for hospital-treated deliberate self-poisoning. *Arch Suicide Res* 2017;21(1):138-154. |
| **Active contact and follow-up group (Telephone)** | |
| 12 | 17. Cedereke M, Monti K, Ojehagen A. Telephone contact with patients in the year after a suicide attempt: does it affect treatment attendance and outcome? A randomised controlled study. *Eur Psychiatry* 2002; **17**(2): 82–91. |
| 13 | 18. Vaiva G, Ducrocq F, Meyer P, et al. Effect of telephone contact on further suicide attempts in patients discharged from an emergency department: randomised controlled study. *BMJ* 2006; **332**(7552): 1241–5. |
| **Active contact and follow-up group (Composite of letter/postcard and telephone)** | |
| 14 | 19. Kapur N, Gunnell D, Hawton K, et al. Messages from Manchester: pilot randomised controlled trial following self-harm. *Br J Psychiatry* 2013; **203**(1): 73–4. |
| **Psychotherapy group** | |
| 15 | 20. Gibbons JS, Butler J, Urwin P, Gibbons JL. Evaluation of a social work service for self-poisoning patients. *Br J Psychiatry* 1978; **133**: 111–8. |
| 16 | 21. Liberman RP, Eckman T. Behavior therapy vs insight-oriented therapy for repeated suicide attempters. *Arch Gen Psychiatry* 1981; **38**(10): 1126–30. |
| 17 | 22. McLeavey BC, Daly RJ, Ludgate JW, Murray CM. Interpersonal problem-solving skills training in the treatment of self-poisoning patients. *Suicide Life Threat Behav* 1994; **24**(4): 382–94. |
| 18 | 23. Guthrie E, Kapur N, Mackway-Jones K, et al. Randomised controlled trial of brief psychological intervention after deliberate self poisoning. *BMJ* 2001; **323**(7305): 135–8. |
| 19 | 24. Raj MA, Kumaraiah V, Bhide AV. Cognitive-behavioural intervention in deliberate self-harm. *Acta Psychiatr Scand* 2001; **104**(5): 340–5. |
| 20 | 25. Brown GK, Ten Have T, Henriques GR, Xie SX, Hollander JE, Beck AT. Cognitive therapy for the prevention of suicide attempts: a randomized controlled trial. *JAMA : the journal of the American Medical Association* 2005; **294**(5): 563–70.  26. Ghahramanlou-Holloway M, Bhar SS, Brown GK, Olsen C, Beck AT. Changes in problem-solving appraisal after cognitive therapy for the prevention of suicide. *Psychol Med* 2012; **42**(6): 1185–93. |
| 21 | 27. Bannan N. Group-based problem-solving therapy in self-poisoning females: A pilot study. *Counsel Psychotherapy Res* 2010; **10**(3): 201–13. |

**Table S1 List of selected trials and publications (continued)**

| **Trial No.** | **Reference No. (see Additional file 11). Authors. Title. Journal year; volume(issue): pages** |
| --- | --- |
| **Psychotherapy group (continued)** | |
| 22 | 28. Ougrin D, Zundel T, Ng A, Banarsee R, Bottle A, Taylor E. Trial of Therapeutic Assessment in London: randomised controlled trial of Therapeutic Assessment versus standard psychosocial assessment in adolescents presenting with self-harm. *Arch Dis Child* 2011; **96**(2): 148–53.  29. Ougrin D, Boege I, Stahl D, Banarsee R, Taylor E. Randomised controlled trial of therapeutic assessment versus usual assessment in adolescents with self-harm: 2-year follow-up. *Arch Dis Child* 2013; **98**(10): 772–6. |
| 23 | 30. Wei S, Liu L, Bi B, et al. An intervention and follow-up study following a suicide attempt in the emergency departments of four general hospitals in Shenyang, China. *Crisis* 2013; **34**(2): 107–15. |
| 24 | 31. Davidson KM, Brown TM, James V, Kirk J, Richardson J. Manual-assisted cognitive therapy for self-harm in personality disorder and substance misuse: a feasibility trial. *Psychiatr Bull (2014)* 2014; **38**(3): 108–11. |
| **Pharmacotherapy group** | |
| 25 | 32. Battaglia J, Wolff TK, Wagner-Johnson DS, Rush AJ, Carmody TJ, Basco MR. Structured diagnostic assessment and depot fluphenazine treatment of multiple suicide attempters in the emergency department. *Int Clin Psychopharmacol* 1999; **14**(6): 361–72. |
| **Miscellaneous group** | |
| 26 | 33. Torhorst A, Moller HJ, Burk F, Kurz A, Wachtler C, Lauter H. The psychiatric management of parasuicide patients: a controlled clinical study comparing different strategies of outpatient treatment. *Crisis* 1987; **8**(1): 53–61. |
| 27 | 34. Waterhouse J, Platt S. General hospital admission in the management of parasuicide. A randomised controlled trial. *Br J Psychiatry* 1990; **156**: 236–42. |
| 28 | 35. Crawford MJ, Csipke E, Brown A, et al. The effect of referral for brief intervention for alcohol misuse on repetition of deliberate self-harm: an exploratory randomized controlled trial. *Psychol Med* 2010; **40**(11): 1821–8. |

See references in Additional file 11.
