# Supplementary material for: Active contact and follow-up interventions to prevent repeat suicide attempts during high-risk periods among patients admitted to emergency departments for suicidal behavior: a systematic review and meta-analysis
Source: BMC Psychiatry. 2019 Jan 25;19:44. doi: 10.1186/s12888-019-2017-7 (PMC6347824; doi:10.1186/s12888-019-2017-7)
Supplement: Supplementary file 3 — Interventions (psychotherapy, pharmacotherapy, and miscellaneous interventions). (DOCX 30 kb) [file 12888_2019_2017_MOESM3_ESM.docx]

**Table S2 Interventions (psychotherapy, pharmacotherapy, and miscellaneous interventions)**

|  | **Intervention 1** | **Intervention 2/Comparison intervention** | **Control (TAU, Placebo)** |
| --- | --- | --- | --- |
| **Psychotherapy group** | | | |
| Gibbons et al., 1978^20^ | Problem-solving approach | - | TAU: routine service: referral back to a GP, psychiatric referral, or other referral |
| Liberman et al., 1981^21^ | Inpatient treatment with behavioral therapy followed by individual and group therapy plus aftercare at a community mental health center or with private therapists | Inpatient treatment with insight-oriented therapy followed by individual and group therapy plus aftercare at a community mental health center or with private therapists | - |
| McLeavey et al., 1994^22^ | Problem-solving approach | Brief problem-oriented approach | - |
| Guthrie et al., 2001^23^ | Psychodynamic interpersonal therapy | - | TAU: an assessment, and if necessary, referral to a psychiatric outpatient department or addiction services, or advised to consult their own GPs |
| Raj et al., 2001^24^ | Cognitive–behavioral method to enhance compliance | Routine medical treatment plus provision of therapist contact information, and contact by letters twice | - |
| Brown et al., 2005^25^; Ghahramanlow-Holloway et al., 2012^26^ | Cognitive therapy | - | TAU: care from clinicians in the community and referral services from the study case manager, and contact from case manager |
| Bannan, 2010^27^ | Problem-solving approach | - | TAU: standard individual therapy in the outpatient department or day hospital |
| Ougrin et al., 2011^28^, 2013^29^ | Therapeutic assessment: a brief intervention based on cognitive–analytic therapy | - | TAU: standard psychosocial assessment |
| Wei et al., 2013^30^ | Cognitive therapy | Telephone intervention | TAU: all patients in the 3 groups received psychotropic medication if necessary |
| Davidson et al., 2014^31^ | Manual-assisted cognitive therapy |  | TAU: a referral to a community mental health  team |

**Table S2 Interventions (psychotherapy, pharmacotherapy, and miscellaneous type interventions) (continued)**

|  | **Intervention 1** | **Intervention 2/Comparison intervention** | **Control (TAU, Placebo)** |
| --- | --- | --- | --- |
| **Pharmacotherapy group** | | | |
| Battaglia et al., 1999^32^ | Low dose of depot fluphenazine | Ultra-low dose of depot fluphenazine | - |
| **Miscellaneous group** | | | |
| Torhorst et al., 1987^33^ | Continuity of care: therapy with the same therapist who assessed the patient in hospital after a suicide attempt | Change of care: therapy with a different therapist | - |
| Waterhouse et al., 1990^34^ | Admission to a general hospital, and advice to contact their GP after discharge from hospital, if needed | Discharge home from the casualty department with advice to contact their GP, if needed | - |
| Crawford et al., 2010^35^ | Provision of an appointment card with alcohol nurse specialist and a health information leaflet | Provision of a health information leaflet | - |

Abbreviations: TAU, treatment as usual.

See references in Additional file 11.
