# Supplementary material for: Active contact and follow-up interventions to prevent repeat suicide attempts during high-risk periods among patients admitted to emergency departments for suicidal behavior: a systematic review and meta-analysis
Source: BMC Psychiatry. 2019 Jan 25;19:44. doi: 10.1186/s12888-019-2017-7 (PMC6347824; doi:10.1186/s12888-019-2017-7)
Supplement: Supplementary file 4 — Results (psychotherapy, pharmacotherapy, and miscellaneous interventions). (DOCX 34 kb) [file 12888_2019_2017_MOESM4_ESM.docx]

**Table S3 Results (psychotherapy, pharmacotherapy, and miscellaneous interventions)**

|  | | **Re-attempts** |  | |  | **Deaths** |  |
| --- | --- | --- | --- | --- | --- | --- | --- |
|  | | **No. of patients with re-attempts/No. of patients in each group analysis** | **No. of re-attempts/No. of patients in each group analysis** | |  | **No. of any-cause deaths/No. of patients in each group analysis** | **No. of suicidal deaths/No. of patients in each group analysis** |
| **Psychotherapy group** | | | | | | | |
| Gibbons et al., 1978^20^ | | E: 27/200; C: 29/200 | - | |  | - | - |
| Liberman et al., 1981^21^ | | E1: 3/12; E2: 2/12 | E1: 5/12; E2: 6/12 | |  | - | - |
| McLeavey et al., 1994^22^ | | ^a^E1: 2/19; E2: 5/20 | - | |  | - | - |
| Guthrie et al., 2001^23^ | | E: 5/58; C: 17/61 | - | |  | - | E: 0/58; C: 0/61 |
| Raj et al., 2001^24^ | | - | - | |  | - | - |
| Brown et al., 2005^25^; Ghahramanlow-Holloway et al., 2012^26^ | | E: 13/45; C: 23/40 | - | |  | E: 1/45; C: 3/40 | E: 0/45; C: 1/40 |
| Bannan, 2010^27^ | | E: 0/9; C: 0/9 | - | |  | - | - |
| Ougrin et al., 2011^28^, 2013^29^ * | | No. of patients with re-attempts at 12 mo not shown  E: 7/35; C: 9/34 at 24 mo | - | |  | - | - |
| Wei et al., 2013^30^ | | E1: 1/25; E2: 1/36; C: 5/27 | - | |  | E1: 1/25; E2: 0/36; C: 0/27 | - |
| Davidson et al., 2014^31^ * | | - | - | |  | - | - |
| **Pharmacotherapy group** | | | | | | | |
| Battaglia et al., 1999^32^ | | The numbers of patients with re-attempts were not shown (p = .15 for frequency of "serious" self-harm/mo between E1 and E2) | - | |  | - | - |
| **Miscellaneous group** | | | | | | | |
| Torhorst et al., 1987^33^ | E1: 12/65; E2: 4/68 | | | - |  | - | - |
| Waterhouse et al., 1990^34^ | - | | | - |  | - | - |
| Crawford et al., 2010^35^ | E1: 7/51; E2: 11/52 | | | - |  | E1: 1/51; E2: 0/52 | - |

Abbreviations: E, experimental intervention group; C, control group.

^a^Five patients who dropped out of treatment were not assessed.

We referred to and modified data from a previous paper by Inagaki et al.[^1^](#_ENREF_1), and we reviewed newly published studies and added new data* to the present table.

See references in Additional file 11.
