# Supplementary material for: Active contact and follow-up interventions to prevent repeat suicide attempts during high-risk periods among patients admitted to emergency departments for suicidal behavior: a systematic review and meta-analysis
Source: BMC Psychiatry. 2019 Jan 25;19:44. doi: 10.1186/s12888-019-2017-7 (PMC6347824; doi:10.1186/s12888-019-2017-7)
Supplement: Supplementary file 5 — Subjects. (DOCX 84 kb) [file 12888_2019_2017_MOESM5_ESM.docx]

**Table S4 Subjects**

|  | **Inclusion** | **Exclusion** | **No. of patients randomly allocated to each group** | | |
| --- | --- | --- | --- | --- | --- |
|  |  |  | **Intervention 1** | **Intervention 2/Comparison** | **Control (TAU, Placebo)** |
| **Active contact and follow-up group (Intensive care plus outreach)** | | | | | |
| Allard et al., 1992[^2^](#_ENREF_2) |  | High risk of suicide | 76 | - | 74 |
| Van Heeringen et al., 1995[^3^](#_ENREF_3) |  |  | 258 | - | 258 |
| van der Sande et al., 1997[^4^](#_ENREF_4) |  |  | 140 | - | 134 |
| Morthorst et al., 2012[^5^](#_ENREF_5) |  | Specific psychiatric disorders, psychosis, or requiring immediate psychiatric treatment | 123 | - | 120 |
| Kawanishi et al., 2014[^6^](#_ENREF_6) * | Axis I psychiatric disorder by the DSM-IV-TR as primary diagnosis |  | 460 | - | 456 |
| Hatcher et al., 2015[^7^](#_ENREF_7) * |  |  | 737 |  | 737 |
| **Active contact and follow-up group (Brief intervention and contact)** | | | | | |
| Fleischmann et al., 2008[^8^](#_ENREF_8);  Bertolote et al., 2010[^9^](#_ENREF_9) |  |  | 922 | - | 945 |
| Mousavi et al., 2014[^10^](#_ENREF_10) * | Poisoning with repetition |  | 69 |  | 70 |
| **Active contact and follow-up group (Letter or postcard)** | | | | | |
| Carter et al., 2005[^11^](#_ENREF_11); 2007[^12^](#_ENREF_12); 2013[^13^](#_ENREF_13) | Poisoning |  | 378 | - | 394 |
| Beautrais et al., 2010[^14^](#_ENREF_14) |  |  | 153 | - | 174 |
| Hassanian-Moghaddam et al., 2011[^15^](#_ENREF_15); 2015[^16^](#_ENREF_16) * | Poisoning | Psychosis | 1150 | - | 1150 |
| **Active contact and follow-up group (Telephone)** | | | | | |
| Cedereke et al., 2002[^17^](#_ENREF_17) |  |  | 107 | - | 109 |
| Vaiva et al., 2006[^18^](#_ENREF_18) | Poisoning |  | 147 | 146 | 312 |
| **Active contact and follow-up group (Composite of letter/postcard and telephone)** | | | | | |
| Kapur et al., 2013[^19^](#_ENREF_19) |  | Psychosis | 33 | - | 33 |

**Table S4 Subjects (continued)**

|  | **Inclusion** | **Exclusion** | **No. of patients randomly allocated to each group** | | |
| --- | --- | --- | --- | --- | --- |
|  |  |  | **Intervention 1** | **Intervention 2/Comparison** | **Control (TAU, Placebo)** |
| **Psychotherapy group** | | | | | |
| Gibbons et al., 1978[^20^](#_ENREF_20) | Poisoning | Psychiatric illness requiring immediate psychiatric treatment, or high risk of suicide | 200 | - | 200 |
| Liberman et al., 1981[^21^](#_ENREF_21) | Repetition | Addicted to alcohol or other drugs, or psychosis | 12 Insight-oriented therapy | 12 Behavioral therapy | - |
| McLeavey et al., 1994[^22^](#_ENREF_22) | Poisoning | Mental retardation, psychosis, requiring immediate psychiatric treatment, or high risk of suicide | 19 | 20 | - |
| Guthrie et al., 2001[^23^](#_ENREF_23) | Poisoning | Requiring immediate psychiatric treatment | 58 | - | 61 |
| Raj et al., 2001[^24^](#_ENREF_24) | Poisoning in patients with anxiety, depression, or adjustment disorders | Psychiatric disorders other than inclusion criteria, psychosis, or score of less than 20 on the Mini-Mental State Examination | 20 | 20 | - |
| Brown et al., 2005[^25^](#_ENREF_25); Ghahramanlow-Holloway et al., 2012[^26^](#_ENREF_26) |  | Medical disorder that would prevent participation in an outpatient clinical trial | 60 | - | 60 |
| Bannan, 2010[^27^](#_ENREF_27) | Repetition | Addiction or psychosis | 9 | - | 9 |
| Ougrin et al., 2011[^28^](#_ENREF_28), 2013[^29^](#_ENREF_29) * |  | Learning disability, requiring immediate psychiatric treatment, or high risk of suicide | 35 | - | 35 or 34 |
| Wei et al., 2013[^30^](#_ENREF_30) |  |  | 82 | 80 | 77 |
| Davidson et al., 2014[^31^](#_ENREF_31) * | Personality disorder |  | 14 |  | 6 |
| **Pharmacotherapy group** | | | | | |
| Battaglia et al., 1999[^32^](#_ENREF_32) | Repetition | Psychosis | 30 | 28 | - |
| **Miscellaneous group** | | | | | |
| Torhorst et al., 1987[^33^](#_ENREF_33) |  | Psychosis | 68 | 73 | - |
| Waterhouse et al., 1990[^34^](#_ENREF_34) |  | Current psychiatric inpatients requiring immediate psychiatric treatment | 38 | 39 | - |
| Crawford et al., 2010[^35^](#_ENREF_35) | Alcohol misuse | Contact with alcohol misuse services | 51 | 52 | - |

Abbreviations: TAU, treatment as usual. We referred to and modified data from a previous paper by Inagaki et al.[^1^](#_ENREF_1), and we reviewed newly published studies and added new data* to the present table.

See references in Additional file 11.
