# Supplementary material for: Active contact and follow-up interventions to prevent repeat suicide attempts during high-risk periods among patients admitted to emergency departments for suicidal behavior: a systematic review and meta-analysis
Source: BMC Psychiatry. 2019 Jan 25;19:44. doi: 10.1186/s12888-019-2017-7 (PMC6347824; doi:10.1186/s12888-019-2017-7)
Supplement: Supplementary file 6 — Adherence to intervention and follow-up rate. (DOCX 84 kb) [file 12888_2019_2017_MOESM6_ESM.docx]

**Table S5 Adherence to intervention and follow-up rate**

|  | **Adherence to intervention** | **Follow-up rate (deaths and suicide re-attempts)** | **Max. period** |
| --- | --- | --- | --- |
| **Active contact and follow-up group (Intensive care plus outreach)** | | | |
| Allard et al., 1992[^2^](#_ENREF_2) | 21 of 61 in E who could be assessed for repeat attempts received complete intervention | Re-attempts: 63/76 in E and 63/74 in C  Deaths: 76/76 in E and 74/74 in C | 24 mo |
| Van Heeringen et al., 1995[^3^](#_ENREF_3) | - | Re-attempts & Deaths: 196/258 in E and 195/258 in C | 12 mo |
| van der Sande et al., 1997[^4^](#_ENREF_4) | - | Re-attempts: 140/140 in E and 134/134 in C | 12 mo |
| Morthorst et al., 2012[^5^](#_ENREF_5) | The offered service was flexible. 5/123 in E did not receive the allocated intervention because they withdrew from the study | Re-attempts (medical records): 123/123 in E and 120/120 in C  Re-attempts (self-reports): 95/123 in E and 74/120 in C  Deaths: 123/123 in E and 120/120 in C | 12 mo |
| Kawanishi et al., 2014[^6^](#_ENREF_6) * | 320/460 participants were contacted at least 7 times | Re-attempts: 325/460 in E and 313/454 in C  Deaths: 451/460 in E and 441/454 in C | 60 mo |
| Hatcher et al., 2015[^7^](#_ENREF_7) * | 327/737 consented to receive intervention in E | Hospital repetitions: 737/737 in E and 737/737 in C  Self-report repetitions: 267/737 in E and 312/737 in C | 12 mo |
| **Active contact and follow-up group (Brief intervention and contact)** | | | |
| Fleischmann et al., 2008[^8^](#_ENREF_8); Bertolote et al., 2010[^9^](#_ENREF_9) | - | Re-attempts: 863/922 in E and 800/945  Deaths: 872/922 in E and 827/945 in C | 18 mo |
| Mousavi et al., 2014[^10^](#_ENREF_10) * | - | - | 6 mo |
| **Active contact and follow-up group (Letter or postcard)** | | | |
| Carter et al., 2005[^11^](#_ENREF_11); 2007[^12^](#_ENREF_12); 2013[^13^](#_ENREF_13) | Among 379 in E, 76 refused the intervention, 1 missed the intervention, and 32 did not receive the full intervention | Re-attempts & Deaths: 378/378 in E and 394/394 in C | 60 mo |
| Beautrais et al., 2010[^14^](#_ENREF_14) | - | Re-attempts: 153/153 in E and 174/174 in C | 12 mo |
| Hassanian-Moghaddam et al., 2011[^15^](#_ENREF_15); 2015[^16^](#_ENREF_16) * | - | Re-attempts: 1043/1150 in E and 1070/1150 in C at 12 mo  Deaths: 1150/1150 in E and 1150/1150 in C at 12 mo  Re-attempts: 997/1150 in E and 1004/1150 in C at 24 mo  Deaths: 1150/1150 in E and 1150/1150 in C at 24 mo | 24 mo |
| **Active contact and follow-up group (Telephone)** | | | |
| Cedereke et al., 2002[^17^](#_ENREF_17) | 83/107 in E received at least 1 intervention | Re-attempts: 83/107 in E and 89/109 in C  Deaths: 107/107 in E and 109/109 in C | 12 mo |
| Vaiva et al., 2006[^18^](#_ENREF_18) | 107/147 in E1 and 97/146 in E2 received the intervention | Re-attempts & Deaths: 147/147 in E1, 146/146 in E2, and 312/312 in C | 13 mo |
| **Active contact and follow-up group (Composite of letter/postcard and telephone)** | | | |
| Kapur et al., 2013[^19^](#_ENREF_19) | 1/33 in E discontinued intervention | Re-attempts: 33/33 in E and 32/33 in C; Deaths: 33/33 in E and 32/33 in C | 12 mo |

**Table S5 Adherence to intervention and follow-up rate (continued)**

|  | **Adherence to intervention** | **Follow-up rate (death and suicide re-attempts)** | **Max. period** |
| --- | --- | --- | --- |
| **Psychotherapy group** | | | |
| Gibbons et al., 1978[^20^](#_ENREF_20) | Contact lasted for 12 wk or less in 2/3 of the cases | Re-attempts: 200/200 in E and 200/200 in C | 12 mo |
| Liberman et al., 1981[^21^](#_ENREF_21) | - | Re-attempts: 12/12 in E and 12/12 in E2 | 24 mo |
| McLeavey et al., 1994[^22^](#_ENREF_22) | 2/19 in E1 and 3/20 in E2 dropped out from intervention | Re-attempts: 19/19 in E1 and 20/20 in E2 | 12 mo |
| Guthrie et al., 2001[^23^](#_ENREF_23) | 35/58 in E completed the 4 intervention sessions | Re-attempts & Deaths: 58/58 in E and 61/61 in C | 6 mo |
| Raj et al., 2001[^24^](#_ENREF_24) | - | - | 3 mo |
| Brown et al., 2005[^25^](#_ENREF_25); Ghahramanlow-Holloway et al., 2012[^26^](#_ENREF_26) | 30/60 in E completed the 10 intervention sessions | Re-attempts & Deaths: 45/60 in E and 40/60 in C | 18 mo |
| Bannan, 2010[^27^](#_ENREF_27) | - | Re-attempts: 9/9 in E and 9/9 in C | 2 mo |
| Ougrin et al., 2011[^28^](#_ENREF_28), 2013[^29^](#_ENREF_29) * | 35/35 in E received the allocated intervention at 3 mo | No data at 3 mo  Re-attempts: 35/35 in E and 34/35 in C at 24 mo | 24 mo |
| Wei et al., 2013[^30^](#_ENREF_30) | 5/82 in E1 and 8/60 in E2 received the intervention | Re-attempts & Deaths: 25/82 in E1, 36/80 in E2, and 27/77 in C | 12 mo |
| Davidson et al., 2014[^31^](#_ENREF_31) * | 9/14 in E received 4 or more sessions (mean 5.4, SD = 1.3), 2/14 never attended and a further 3/14 people attended between 1 and 3 sessions | Deaths: 10/14 in E and 4/6 in C, (no re-attempts) | 3 mo |
| **Pharmacotherapy group** | | | |
| Battaglia et al., 1999[^32^](#_ENREF_32) | 12/30 in E1 and 13/28 in E2 completed the 6-mo treatment | Re-attempts: 27/30 in E1 and 26/28 in E2 | 6 mo |
| **Miscellaneous group** | | | |
| Torhorst et al., 1987[^33^](#_ENREF_33) | 32/68 in E1 and 20/73 in E2 completed more than 4–12 sessions | Re-attempts: 65/68 in E1 and 68/73 in E2 | 12 mo |
| Waterhouse et al., 1990[^34^](#_ENREF_34) | - | - | - |
| Crawford et al., 2010[^35^](#_ENREF_35) | 24/51 in E received the intervention | Re-attempts & Deaths: 51/51 in E1 and 52/52 in E2 | 6 mo |

Abbreviations: E, experimental intervention group; C, control group. We referred to and modified data from a previous paper by Inagaki et al.[^1^](#_ENREF_1), and we reviewed newly published studies and added new data* to the present table.

See references in Additional file 11.
