# Supplementary material for: Active contact and follow-up interventions to prevent repeat suicide attempts during high-risk periods among patients admitted to emergency departments for suicidal behavior: a systematic review and meta-analysis
Source: BMC Psychiatry. 2019 Jan 25;19:44. doi: 10.1186/s12888-019-2017-7 (PMC6347824; doi:10.1186/s12888-019-2017-7)
Supplement: Supplementary file 7 — Measure of suicidal behaviors. (DOCX 87 kb) [file 12888_2019_2017_MOESM7_ESM.docx]

**Table S6 Measure of suicidal behaviors**

|  | **Telephone** | **Interview** | **Medical record** | **GPs** | **Relatives/friends** | **Death register/**  **Coroner's office** |
| --- | --- | --- | --- | --- | --- | --- |
| **Active contact and follow-up group (Intensive care plus outreach)** | | | | | | |
| Allard et al., 1992[^2^](#_ENREF_2) | Attempt |  | Attempt |  | Attempt | Death |
| Van Heeringen et al., 1995[^3^](#_ENREF_3) |  | Attempt |  | Attempt | Attempt | Death |
| van der Sande et al., 1997[^4^](#_ENREF_4) |  |  | Attempt | Death |  | Death |
| Morthorst et al., 2012[^5^](#_ENREF_5) | Attempt |  | Attempt/Death |  |  | Death |
| Kawanishi et al., 2014[^6^](#_ENREF_6) * |  | Attempt/Death |  |  |  | Death |
| Hatcher et al., 2015[^7^](#_ENREF_7) ^a^ * | Attempt |  | Attempt |  |  |  |
| **Active contact and follow-up group (Brief intervention and contact)** | | | | | | |
| Fleischmann et al., 2008[^8^](#_ENREF_8);  Bertolote et al., 2010[^9^](#_ENREF_9) | Attempt | Attempt |  |  | Death |  |
| Mousavi et al., 2014[^10^](#_ENREF_10) * |  |  |  |  |  |  |
| **Active contact and follow-up group (Letter or postcard)** | | | | | | |
| Carter et al., 2005[^11^](#_ENREF_11); 2007[^12^](#_ENREF_12); 2013[^13^](#_ENREF_13) |  |  | Attempt |  |  | Death |
| Beautrais et al., 2010[^14^](#_ENREF_14) |  |  | Attempt |  |  |  |
| Hassanian-Moghaddam et al., 2011[^15^](#_ENREF_15); 2015[^16^](#_ENREF_16) ^b^ * | Attempt | Attempt | Attempt |  |  | Death |
| **Active contact and follow-up group (Telephone)** | | | | | | |
| Cedereke et al., 2002[^17^](#_ENREF_17) |  | Attempt | Attempt |  |  | Death |
| Vaiva et al., 2006[^18^](#_ENREF_18) | Attempt |  | Attempt | Attempt |  | Death |
| **Active contact and follow-up group (Composite of letter/postcard and telephone)** | | | | | | |
| Kapur et al., 2013[^19^](#_ENREF_19) ^a^ |  |  | Attempt |  |  |  |

**Table S6 Methods to measure suicidal behaviors (continued)**

|  | **Telephone** | **Interview** | **Medical record** | **GPs** | **Relatives/friends** | **Death register/**  **Coroner's office** |
| --- | --- | --- | --- | --- | --- | --- |
| **Psychotherapy group** | | | | | | |
| Gibbons et al., 1978[^20^](#_ENREF_20) |  | Attempt | Attempt | Attempt |  |  |
| Liberman et al., 1981[^21^](#_ENREF_21) |  | Attempt |  |  |  |  |
| McLeavey et al., 1994[^22^](#_ENREF_22) |  |  | Attempt | Attempt |  |  |
| Guthrie et al., 2001[^23^](#_ENREF_23) ^a^ |  | Attempt | Attempt |  |  |  |
| Raj et al., 2001[^24^](#_ENREF_24) ^c^ |  |  |  |  |  |  |
| Brown et al., 2005[^25^](#_ENREF_25); Ghahramanlow-Holloway et al, 2012[^26^](#_ENREF_26) ^a^ |  | Attempt |  |  |  |  |
| Bannan, 2010[^27^](#_ENREF_27) ^d^ |  |  |  |  |  |  |
| Ougrin et al., 2011[^28^](#_ENREF_28), 2013[^29^](#_ENREF_29) ^c^ * |  |  | Attempt |  |  |  |
| Wei et al., 2013[^30^](#_ENREF_30) ^a^ | Attempt |  |  |  |  |  |
| Davidson et al., 2014[^31^](#_ENREF_31) ^a^ * |  |  |  |  |  |  |
| **Pharmacotherapy group** | | | | | | |
| Battaglia et al., 1999[^32^](#_ENREF_32) | Attempt | Attempt |  |  |  |  |
| **Miscellaneous group** | | | | | | |
| Torhorst et al., 1987[^33^](#_ENREF_33) ^d^ |  |  |  |  |  |  |
| Waterhouse et al., 1990[^34^](#_ENREF_34) ^c^ |  |  |  |  |  |  |
| Crawford et al., 2010[^35^](#_ENREF_35) ^a^ |  |  | Attempt |  |  |  |

^a^The method of measuring suicidal death was not clear.

^b^Follow-up was by telephone for all but three participants, who were interviewed in person.

^c^Suicide behaviors were not measured.

^d^The method of measuring a suicide attempt was not clear.

We referred to and modified data from a previous paper by Inagaki et al.[^1^](#_ENREF_1), and we reviewed newly published studies and added new data* to the present table.

See references in Additional file 11.
