# Supplementary material for: Active contact and follow-up interventions to prevent repeat suicide attempts during high-risk periods among patients admitted to emergency departments for suicidal behavior: a systematic review and meta-analysis
Source: BMC Psychiatry. 2019 Jan 25;19:44. doi: 10.1186/s12888-019-2017-7 (PMC6347824; doi:10.1186/s12888-019-2017-7)
Supplement: Supplementary file 8 — List of psychometric measures. a: Suicidal ideation. b: Hopelessness. c: Sense of belonging. d: Depression, anxiety, and general mental health. e: Alcohol-related problems. f: Quality of life and global functioning. g: Problem solving. h: Others. (DOCX 359 kb) [file 12888_2019_2017_MOESM8_ESM.docx]

**Table S7a List of psychometric measures (suicidal ideation)**

|  | **Validation** | **Baseline** | **1 wk/2 wk** | **1 mo** | **6 wk/2 mo** | **3 mo** | **4 mo** | **6 mo** | **9 mo** | **12 mo** | **24 mo** | **60 mo** |
| --- | --- | --- | --- | --- | --- | --- | --- | --- | --- | --- | --- | --- |
|  |  | **Data are shown as "mean [SD] (n)," "N [%] (n)," or “median [IQR] (n)”** | | | | | | | | | | |
| **Scale for Suicide Ideation (SSI: Beck, 1979)** | | | | | | | | | | | | |
| Suicide Ideation[^17^](#_ENREF_17)^,^[^23^](#_ENREF_23)^,^[^24^](#_ENREF_24)^,^[^26^](#_ENREF_26)^,^[^27^](#_ENREF_27)^,^[^31^](#_ENREF_31) | R  V  P | E: 15.9 [9.9] (58); C: 14.3 [10.8] (61) [^23^](#_ENREF_23)  E: 19.05 [5.51] (20); C: 18.20 [5.65] (20)[^24^](#_ENREF_24)  E: 12.1 [6.3] (9); C: 15.8 [8.8] (9)[^27^](#_ENREF_27)  E: 21 [8.41] (14);  C: 24.33 [5.16] (6)[^31^](#_ENREF_31)  No data[^26^](#_ENREF_26) |  | E: 8.4 [8.7] (107); C: 5.3 [6.7] (109)[^17^](#_ENREF_17)  E: 10.3 [8.6] (no data); C: 12.4 [9.9] (no data) after intervention[^23^](#_ENREF_23) | E: 5.8 [8.3] (9);  C: 12.6 [9.0] (9) after intervention[^27^](#_ENREF_27) | E: 15.00 [7.79] (20); C: 2.75 [6.09] (20)[^24^](#_ENREF_24) ^a^  E: 12.36 [12.48] (11); C: 26.5 [1.92] (4)[^31^](#_ENREF_31) | E: 1.3 [3.0] (9); C: 11.0 [10.2] (9) 2 mo after intervention[^27^](#_ENREF_27) | E: 7.9 [8.6] (no data); C: 12.8 [10.4] (no data)[^23^](#_ENREF_23) |  | E: 5.8 [7.8] (no data); C: 4.0 [6.2] (no data)[^17^](#_ENREF_17) |  |  |

**Table S7a List of psychometric measures (suicidal ideation) (continued)**

|  | **Validation** | **Baseline** | **1 wk/2 wk** | **1 mo** | **6 wk/2 mo** | **3 mo** | **4 mo** | **6 mo** | **9 mo** | **12 mo** | **24 mo** | **60 mo** |
| --- | --- | --- | --- | --- | --- | --- | --- | --- | --- | --- | --- | --- |
|  |  | **Data are shown as "mean [SD] (n)," "N [%] (n)," or “median [IQR] (n)”** | | | | | | | | | | |
| **Scale for Suicide Ideation (SSI: Beck, 1979) (Number of patients with scores >0)** | | | | | | | | | | | | |
| Number of patients with suicide Ideation[^25^](#_ENREF_25)^,^[^30^](#_ENREF_30) | R  V  P | E: 39 [65.0%] (60); C: 39 [65.0%] (60)[^25^](#_ENREF_25)  E1: 53 [64.6%] (82); E2: 56 [70.0%] (80 were assessed); C: 57 [74.0%] (77)[^30^](#_ENREF_30) |  | E: 24 [44.4%] (54); C: 26 54 [46.4%] (56)[^25^](#_ENREF_25) |  | E: 20 [38.5%] (52); C: 24 [44.4%] (54)[^25^](#_ENREF_25)  E1: 33 [40.2% of 82] (55 were assessed); E2: 37 [46.3% of 80] (56 were assessed); C: 32 [41.6% of 77] (61 were assessed)[^30^](#_ENREF_30) |  | E: 12 [24.0%] (50); C: 16 [30.8%] (52)[^25^](#_ENREF_25)  E1: 30 [36.6% of 82] (34 were assessed); E2: 26 [32.5% of 80] (41 were assessed); C: 24 [31.2% of 77] (40 were assessed)[^30^](#_ENREF_30) |  | E: 10 [20.4%] (49); C: 12 [24.5%] (49)[^25^](#_ENREF_25)  E1: 30 [36.6% of 82] (24 were assessed); E2: 24 [30.0% of 80] (36 were assessed); C: 25 [32.5% of 77] (27 were assessed)[^30^](#_ENREF_30) | E: 7 [15.6%] (45); C: 9 [22.5%] (40) in 18 mo^[25](#_ENREF_25)^ |  |

Abbreviations: wk, week/weeks; mo, month/months; E, experimental intervention group; C, control group.

The Validation column shows whether the psychological measure had been previously shown to have reliability, validity, and predictability. R: Previous evidence for reliability; V: Previous evidence for validity; P: Previous evidence for predictability.

^a^Pre–post difference.

See references in Additional file 11.

**Table S7b List of psychometric measures (hopelessness)**

|  | **Validation** | **Baseline** | **1 wk/2 wk** | **1 mo** | **6 wk/2 mo** | **3 mo** | **4 mo** | **6 mo** | **9 mo** | **12 mo** | **24 mo** | **60 mo** |
| --- | --- | --- | --- | --- | --- | --- | --- | --- | --- | --- | --- | --- |
|  |  | **Data are shown as "mean [SD] (n)," "N [%] (n)," or “median [IQR] (n)”** | | | | | | | | | | |
| **Beck Hopelessness Scale (BHS)** | | | | | | | | | | | | |
| Hopelessness[^4^](#_ENREF_4)^,^[^6^](#_ENREF_6)^,^[^7^](#_ENREF_7)^,^[^9^](#_ENREF_9)^,^[^22^](#_ENREF_22)^,^[^24-27^](#_ENREF_24)^,^[^34^](#_ENREF_34) | R  V  P | E: 9.6 [5.9] (140); C: 10.5 [6.0] (134)[^4^](#_ENREF_4)  E: 11.1 [6.2] (300); C: 11.3 [6.1] (331)[^7^](#_ENREF_7)  E: 6.12 [4.61] (19); C: 4.35 [4.39] (20)[^22^](#_ENREF_22)  E: 11.25 [5.54] (20); C: 8.55 [3.55] (20)[^24^](#_ENREF_24)  E: 11.48 [5.45] (60); C: 11.81 [6.25] (60)[^25^](#_ENREF_25)  E: 13.7 [4.4] (9); C: 13.3 [3.4] (9)[^27^](#_ENREF_27) |  | E: 9.09 [5.91] (54 were assessed); C: 8.71 [6.59] (56 were assessed)[^25^](#_ENREF_25) | E: 10.8 [3.3] (9); C: 12.8 [3.5] (9) after intervention[^27^](#_ENREF_27) | E: 8.4 [6.7] (211); C: 9.4 [6.4] (243)[^7^](#_ENREF_7)  E: 5.05 [7.01] (20); C: 0.25 [3.7] (20)[^24^](#_ENREF_24) ^a^  E: 7.45 [4.99] (52 were assessed); C: 9.06 [6.98] (54 were assessed)[^25^](#_ENREF_25) | E: 7.7 [3.0] (9); C: 12.8 [4.0] (9) 2 mo after intervention[^27^](#_ENREF_27) | E: 5.57 [4.47] (50 were assessed); C: 8.21 [6.96] (52 were assessed)[^25^](#_ENREF_25) |  | E: 6.1 [5.0] (140); C: 7.5 [5.9] (134)[^4^](#_ENREF_4)  E: 8.3 [6.3] (210); C: 8.4 [6.4] (233)[^7^](#_ENREF_7)  E: 4.94 [8.38] (17); C: 4.44 [6.21] (16)[^22^](#_ENREF_22)  E: 6.57 [5.76] (49 were assessed); C: 8.22 [6.77] (49 were assessed)[^25^](#_ENREF_25) | E: 6.07 [5.28] (45 were assessed); C: 7.24 [6.35] (40 were assessed) in 18 mo^[25](#_ENREF_25)^ |  |

**Table S7b List of psychometric measures (hopelessness) (continued)**

|  | **Validation** | **Baseline** | **1 wk/2 wk** | **1 mo** | **6 wk/2 mo** | **3 mo** | **4 mo** | **6 mo** | **9 mo** | **12 mo** | **24 mo** | **60 mo** |
| --- | --- | --- | --- | --- | --- | --- | --- | --- | --- | --- | --- | --- |
|  |  | **Data are shown as "mean [SD] (n)," "N [%] (n)," or “median [IQR] (n)”** | | | | | | | | | | |
| **Beck Hopelessness Scale (continued)** | | | | | | | | | | | | |
| (continued)  Hopelessness [^4^](#_ENREF_4)^,^[^6^](#_ENREF_6)^,^[^7^](#_ENREF_7)^,^[^9^](#_ENREF_9)^,^[^22^](#_ENREF_22)^,^[^24-27^](#_ENREF_24)^,^[^34^](#_ENREF_34) | R  V  P | E: 13.7 [4.4] (9); C: 13.3 [3.4] (9)[^27^](#_ENREF_27)  No data[^6^](#_ENREF_6)^,^[^9^](#_ENREF_9)^,^[^26^](#_ENREF_26) | E: 10.29 [5.68] (25); C: 10.21 [4.97] (33) at 1 wk [^34^](#_ENREF_34)  E: 10.29 [5.68] (25); C: 10.21 [4.97] (33) at 1 wk^[34](#_ENREF_34)^ |  | E: 10.8 [3.3] (9); C: 12.8 [3.5] (9) after intervention[^27^](#_ENREF_27) |  | E: 7.7 [3.0] (9); C: 12.8 [4.0] (9) 2 mo after intervention[^27^](#_ENREF_27) |  |  |  |  |  |
| **Subscales of the Beck Hopelessness Scale** | | | | | | | | | | | | |
| Feelings about the future (F: I)[^24^](#_ENREF_24) | R  V  P | E: 2 [1.78] (20); C: 1 [1.21] (20) |  |  |  | E: 0.75 [1.89] (20); C: -0.25 [1.55] (20) ^a^ |  |  |  |  |  |  |
| Loss of motivation (F: II)[^24^](#_ENREF_24) | R  V  P | E: 5.2 [2.40] (20); C: 4 [2.03] (20) |  |  |  | E: 2.75 [2.97] (20); C: 0.05 [1.91] (20) ^a^ |  |  |  |  |  |  |
| Future expectations (F: III)[^24^](#_ENREF_24) | R  V  P | E: 3.2 [1.36] (20); C: 2.9 [1.02] (20) |  |  |  | E: 1.15 [2.06] (20); C: 0.3 [1.49] (20) ^a^ |  |  |  |  |  |  |

Abbreviations: wk, week/weeks; mo, month/months; E, experimental intervention group; C, control group.

The Validation column shows whether the psychological measure had been previously shown to have reliability, validity, and predictability. R: Previous evidence for reliability; V: Previous evidence for validity; P: Previous evidence for predictability.

^a^Pre–post difference.

See references in Additional file 11.

**Table S7c List of psychometric measures (sense of belonging)**

|  | **Validation** | **Baseline** | **1 wk/2 wk** | **1 mo** | **6 wk/2 mo** | **3 mo** | **4 mo** | **6 mo** | **9 mo** | **12 mo** | **24 mo** | **60 mo** |
| --- | --- | --- | --- | --- | --- | --- | --- | --- | --- | --- | --- | --- |
|  |  | **Data are shown as "mean [SD] (n)," "N [%] (n)," or “median [IQR] (n)”** | | | | | | | | | | |
| **Sense of Belonging Instrument (SOBI)** | | | | | | | | | | | | |
| Sense of belonging[^7^](#_ENREF_7) | R  V  - | E: 46.6 [12.5] (320); C: 46.9 [12.7] (354) |  |  |  | E: 42.1 [13.5] (226); C: 44.6 [12.8] (260) |  |  |  | E: 41.3 [13.3] (211); C: 43.0 [13.2] (234) |  |  |
| **Multigroup Ethnic Identity Measure (MEIM)** | | | | | | | | | | | | |
| Sense of belonging[^7^](#_ENREF_7) | R  V  - | E: 14.6 [4.0] (313); C: 14.1 [4.2] (347) |  |  |  | E: 14.2 [4.4] (220); C: 14.8 [3.9] (254) |  |  |  | E: 14.1 [4.1] (211); C: 14.9 [3.8] (234) |  |  |

Abbreviations: wk, week/weeks; mo, month/months; E, experimental intervention group; C, control group.

The Validation column shows whether the psychological measure had been previously shown to have reliability, validity, and predictability. R: Previous evidence for reliability; V: Previous evidence for validity; P: Previous evidence for predictability.

See references in Additional file 11.

**Table S7d List of psychometric measures (depression, anxiety, and general mental health)**

|  | **Validation** | **Baseline** | **1 wk/2 wk** | **1 mo** | **6 wk/2 mo** | **3 mo** | **4 mo** | **6 mo** | **9 mo** | **12 mo** | **24 mo** | **60 mo** |
| --- | --- | --- | --- | --- | --- | --- | --- | --- | --- | --- | --- | --- |
|  |  | **Data are shown as "mean [SD] (n)," "N [%] (n)," or “median [IQR] (n)”** | | | | | | | | | | |
| **Hospital Anxiety and Depression Scale (HADS)** | | | | | | | | | | | | |
| Total[^24^](#_ENREF_24)^,^[^31^](#_ENREF_31) | R  V  - | E: 20.80 [8.52] (20); C: 18.85 [5.03] (20)[^24^](#_ENREF_24)  E: 28.86 [5.55] (14); C: 32.5 [2.59] (6)[^31^](#_ENREF_31) |  |  |  | E: 7.75 [5.66] (20); C: -0.45 [4.6] (20)[^24^](#_ENREF_24) ^a^  E: 21.73 [9.86] (11); C: 33.5 [2.38] (4)[^31^](#_ENREF_31) |  |  |  |  |  |  |
| Depression[^7^](#_ENREF_7)^,^[^24^](#_ENREF_24) | R  V  - | E: 9.9 [2.0] (320); C: 9.9 [2.1] (352)[^7^](#_ENREF_7)  E: 8.75 [5.24] (20); C: 7.85 [3.54] (20)[^24^](#_ENREF_24) |  |  |  | E: 7.6 [5.2] (224); C: 8.0 [5.0] (256)[^7^](#_ENREF_7)  E: 2.9 [3.26] (20); C: -0.35 [2.68] (20)[^24^](#_ENREF_24) ^a^ |  |  |  | E: 6.8 [4.9] (211); C: 6.5 [5.1] (234)[^7^](#_ENREF_7) |  |  |
| Anxiety[^7^](#_ENREF_7)^,^[^24^](#_ENREF_24) | R  V  - | E: 13.2 [4.2] (320); C: 13.0 [4.7] (351)[^7^](#_ENREF_7)  E: 12.05 [4.67] (20); C: 11.00 [3.57] (20)[^24^](#_ENREF_24) |  |  |  | E: 10.5 [5.2] (224); C: 11.1 [5.1] (255)[^7^](#_ENREF_7)  E: 4.85 [4.04] (20); C: -0.1 [4.01] (20)[^24^](#_ENREF_24) ^a^ |  |  |  | E: 10.6 [4.8] (211); C: 10.1 [5.1] (234)[^7^](#_ENREF_7) |  |  |

**Table S7d List of psychometric measures (depression, anxiety, and general mental health) (continued)**

|  | **Validation** | **Baseline** | **1 wk/2 wk** | **1 mo** | **6 wk/2 mo** | **3 mo** | **4 mo** | **6 mo** | **9 mo** | **12 mo** | **24 mo** | **60 mo** |
| --- | --- | --- | --- | --- | --- | --- | --- | --- | --- | --- | --- | --- |
|  |  | **Data are shown as "mean [SD] (n)," "N [%] (n)," or “median [IQR] (n)”** | | | | | | | | | | |
| **Beck Depression Inventory (BDI)** | | | | | | | | | | | | |
| Depression[^9^](#_ENREF_9)^,^[^20^](#_ENREF_20)^,^[^21^](#_ENREF_21)^,^[^23^](#_ENREF_23)^,^[^25-27^](#_ENREF_25) | R  V  P | E: 17.61 [no data] (200); C: 19.63 [no data] (200)[^20^](#_ENREF_20)  E: 26 [15] (12); C: 25 [9] (12) before intervention[^21^](#_ENREF_21)  E: 30.2 [12.2] (58); C: 28.5 [11.6] (61)[^23^](#_ENREF_23) | E: 14 [12] (12); C: 4 [4] (12) after intervention at day 8[^21^](#_ENREF_21)  E: 12 [13] (12); C: 7 [8] (12) at 2 wk^[21](#_ENREF_21)^ | E: 21.3 [13.1] (no data); C: 22.8 [13.3] (no data) after intervention[^23^](#_ENREF_23) | E: 12 [13] (12);  C: 10 [13] (12) at 6 wk^[21](#_ENREF_21)^ | E: 14 [13] (12); C: 8 [9] (12) [^21^](#_ENREF_21) | E: 11.15 [no data] (74); C: 13.49 [no data] (72)[^20^](#_ENREF_20) | E: 13 [11] (12); C: 4 [6] (12)[^21^](#_ENREF_21)  E: 18.8 [13.5] (no data); C: 23.7 [12.6] (no data)[^23^](#_ENREF_23) | E: 11 [12] (12); C: 8 [10] (12)[^21^](#_ENREF_21) |  |  |  |

**Table S7d List of psychometric measures (depression, anxiety, and general mental health) (continued)**

|  | **Validation** | **Baseline** | **1 wk/2 wk** | **1 mo** | **6 wk/2 mo** | **3 mo** | **4 mo** | **6 mo** | **9 mo** | **12 mo** | **24 mo** | **60 mo** |
| --- | --- | --- | --- | --- | --- | --- | --- | --- | --- | --- | --- | --- |
|  |  | **Data are shown as "mean [SD] (n)," "N [%] (n)," or “median [IQR] (n)”** | | | | | | | | | | |
| **Beck Depression Inventory (BDI) (continued)** | | | | | | | | | | | | |
| (continued)  Depression[^9^](#_ENREF_9)^,^[^20^](#_ENREF_20)^,^[^21^](#_ENREF_21)^,^[^23^](#_ENREF_23)^,^[^25-27^](#_ENREF_25) | R  V  P | E: 32.87 [12.03] (60); C: 31.03 [15.70] (60)[^25^](#_ENREF_25)  E: 35.8 [12.7] (9); C: 34.6 [11.7] (9)[^27^](#_ENREF_27)  No data[^9^](#_ENREF_9)^,^[^26^](#_ENREF_26) |  | E: 21.80 [15.48] (54); C: 21.66 [15.44] (56)[^25^](#_ENREF_25) | E: 22.6 [12.6] (9); C: 26.6 [14.3] (9) after intervention[^27^](#_ENREF_27) | E: 19.96 [14.82] (52); C: 21.19 [14.92] (54)[^25^](#_ENREF_25) | E: 1.3 [3.0] (9); C: 11.0 [10.2] (9) 2 mo after intervention[^27^](#_ENREF_27) | E: 13.82 [12.34] (50); C: 19.33 [15.61] (52)[^25^](#_ENREF_25) |  | E: 13.59 [13.40] (49); C: 18.73 [14.87] (49)[^25^](#_ENREF_25) | E: 14.51 [12.90] (45); C: 18.18 [13.75] (40) in 18 mo^[25](#_ENREF_25)^ |  |
| **Beck Depression Inventory (BDI) (Number of patients with moderate–severe BDI scores)** | | | | | | | | | | | | |
| Number of patients with depression[^21^](#_ENREF_21) | -  -  - | E: 9 [75%] (12); C: 9 [75%] (12) before intervention | E: 0 [0%] (12); C: 6 [50%] (12) after intervention at day 8 |  |  |  |  |  |  |  |  |  |

**Table S7d List of psychometric measures (depression, anxiety, and general mental health) (continued)**

|  | **Validation** | **Baseline** | **1 wk/2 wk** | **1 mo** | **6 wk/2 mo** | **3 mo** | **4 mo** | **6 mo** | **9 mo** | **12 mo** | **24 mo** | **60 mo** |
| --- | --- | --- | --- | --- | --- | --- | --- | --- | --- | --- | --- | --- |
|  |  | **Data are shown as "mean [SD] (n)," "N [%] (n)," or “median [IQR] (n)”** | | | | | | | | | | |
| **Zung Self-rating Depression Scale (SDS)** | | | | | | | | | | | | |
| Depression[^21^](#_ENREF_21) | R  V  - | E: 54 [14] (12); C: 52 [9] (12) before intervention | E: 43 [14] (12); C: 32 [8] (12) after intervention at day 8  E: 42 [15] (12); C: 40 [12] (12) at 2 wk |  | E: 40 [14] (12); C: 38 [15] (12) at 6 wk | E: 44 [17] (12);  C: 37 [11] (12) |  | E: 41 [13] (12); C: 34 [8] (12) | E: 39 [13] (12); C: 37 [13] (12) |  |  |  |
| **Zung Self-rating Depression Scale (SDS) (Number of patients with moderate–severe SDS scores)** | | | | | | | | | | | | |
| Number of patients with depression[^21^](#_ENREF_21) | -  -  - | E: 10 [83%] (12); C: 8 [67%] (12) before intervention | E: 0 [0%] (12); C: 7 [58%] (12) after intervention at day 8 |  |  |  |  |  |  |  |  |  |

**Table S7d List of psychometric measures (depression, anxiety, and general mental health) (continued)**

|  | **Validation** | **Baseline** | **1 wk/2 wk** | **1 mo** | **6 wk/2 mo** | **3 mo** | **4 mo** | **6 mo** | **9 mo** | **12 mo** | **24 mo** | **60 mo** |
| --- | --- | --- | --- | --- | --- | --- | --- | --- | --- | --- | --- | --- |
|  |  | **Data are shown as "mean [SD] (n)," "N [%] (n)," or “median [IQR] (n)”** | | | | | | | | | | |
| **Hamilton Rating Scale for Depression (HAMD)** | | | | | | | | | | | | |
| Depression[^25^](#_ENREF_25)^,^[^30^](#_ENREF_30) | R  V  - | E: 26.88 [10.04] (60); C: 26.08 [10.62] (60)[^25^](#_ENREF_25)  E1: 15.09 [10.14] (82); E2: 17.02 [11.35] (80); C: 16.43 [10.29] (77)[^30^](#_ENREF_30) |  | E: 19.89 [10.88] (54); C: 19.05 [12.65] (56)[^25^](#_ENREF_25) |  | E: 17.40 [11.22] (52); C: 19.33 [11.13] (54)[^25^](#_ENREF_25)  E1: 8.43 [10.45] (55); E2: 7.92 [9.26] (56); C: 7.14 [8.33] (61)[^30^](#_ENREF_30) |  | E: 14.70 [11.05] (50); C: 17.83 [13.27] (52)[^25^](#_ENREF_25)  E1: 7.82 [10.38] (34); E2: 6.01 [8.87] (41); C: 5.85 [8.16] (40)[^30^](#_ENREF_30) |  | E: 15.08 [11.44] (49); C: 16.27 [13.82] (49)[^25^](#_ENREF_25)  E1: 7.26 [10.58] (24); E2: 5.73 [8.71] (36); C: 5.84 [8.23] (27)[^30^](#_ENREF_30) | E: 13.09 [9.96] (45); C: 14.55 [11.64] (40) in 18 mo^[25](#_ENREF_25)^ |  |

**Table S7d List of psychometric measures (depression, anxiety, and general mental health) (continued)**

|  | **Validation** | **Baseline** | **1 wk/2 wk** | **1 mo** | **6 wk/2 mo** | **3 mo** | **4 mo** | **6 mo** | **9 mo** | **12 mo** | **24 mo** | **60 mo** |
| --- | --- | --- | --- | --- | --- | --- | --- | --- | --- | --- | --- | --- |
|  |  | **Data are shown as "mean [SD] (n)," "N [%] (n)," or “median [IQR] (n)”** | | | | | | | | | | |
| **Symptom Checklist (SCL-90)** | | | | | | | | | | | | |
| Anxiety[^4^](#_ENREF_4) | R  V  - | E: 28.1 [20.1] (140); C: 27.2 [11.7] (134) |  |  |  |  |  |  |  | E: 18.6 [9.1] (140); C: 19.9 [9.5] (134) |  |  |
| Depression[^4^](#_ENREF_4) | R  V  - | E: 51.6 [17.0] (140); C: 49.9 [17.8] (134) |  |  |  |  |  |  |  | E: 30.8 [15.9] (140); C: 35.8 [16.2] (134) |  |  |
| Phobic anxiety[^4^](#_ENREF_4) | R  V  - | E: 14.5 [7.1] (140); C: 15.5 [7.8] (134) |  |  |  |  |  |  |  | E: 10.9 [6.2] (140); C: 10.9 [4.5] (134) |  |  |
| Somatization[^4^](#_ENREF_4) | R  V  - | E: 30.7 [11.0] (140); C: 28.3 [12.4] (134) |  |  |  |  |  |  |  | E: 21.8 [10.1] (140); C: 24.1 [11.3] (134) |  |  |
| Obsession–compulsion[^4^](#_ENREF_4) | R  V  - | E: 23.3 [8.7] (140); C: 23.9 [9.5] (134) |  |  |  |  |  |  |  | E: 17.6 [8.4] (140); C: 19.2 [9.1] (134) |  |  |
| Interpersonal sensitivity[^4^](#_ENREF_4) | R  V  - | E: 43.6 [16.4] (140); C: 44.6 [15.7] (134) |  |  |  |  |  |  |  | E: 32.1 [14.6] (140); C: 36.1 [15.0] (134) |  |  |
| Hostility[^4^](#_ENREF_4) | R  V  - | E: 12.7 [6.1] (140); C: 12.9 [6.0] (134) |  |  |  |  |  |  |  | E: 9.3 [3.8] (140); C: 9.8 [3.5] (134) |  |  |
| Sleep disorder[^4^](#_ENREF_4) | R  V  - | E: 9.9 [4.2] (140); C: no data |  |  |  |  |  |  |  | E: 6.6 [3.7] (140); C: 7.1 [3.7] (134) |  |  |

**Table S7d List of psychometric measures (depression, anxiety, and general mental health) (continued)**

|  | **Validation** | **Baseline** | **1 wk/2 wk** | **1 mo** | **6 wk/2 mo** | **3 mo** | **4 mo** | **6 mo** | **9 mo** | **12 mo** | **24 mo** | **60 mo** |
| --- | --- | --- | --- | --- | --- | --- | --- | --- | --- | --- | --- | --- |
|  |  | **Data are shown as "mean [SD] (n)," "N [%] (n)," or “median [IQR] (n)”** | | | | | | | | | | |
| **Symptom Checklist (SCL-90) (continued)** | | | | | | | | | | | | |
| General  Symptom Index[^17^](#_ENREF_17) | R  V  - |  |  | E: 1.07 [0.7] (107); C: 0.96 [0.8] (109)  E: 1.05 [0.74] (no data); C: 1.02 [0.77] (no data) |  |  |  |  |  | E: 0.82 [0.78] (no data); C: 0.88 [0.72] (no data) |  |  |
| **Minnesota Multiphasic Personality Inventory (MMPI), depression subscale** | | | | | | | | | | | | |
| Depression[^21^](#_ENREF_21) | R  V  P | E: 35 [9] (12); C: 34 [6] (12) before intervention | E: 30 [10] (12); C: 21 [7] (12) after intervention at day 8  E: 29 [10] (12); C: 24 [7] (12) at 2 wk |  | E: 28 [10] (12); C: 24 [10] (12) at 6 wk | E: 29 [10] (12); C: 23 [10] (12) |  | E: 28 [10] (12); C: 22 [7] (12) | E: 28 [9] (12); C: 25 [11] (12) |  |  |  |
| **Minnesota Multiphasic Personality Inventory (MMPI), depression subscale (Number of patients with deviant score)** | | | | | | | | | | | | |
| Number of patients with depression[^21^](#_ENREF_21) | -  -  - | E: 11 [92%] (12); C: 9 [75%] (12) before intervention | E: 2 [17%] (12); C: 6 [50%] (12) after intervention at day 8 |  |  |  |  |  |  |  |  |  |

**Table S7d List of psychometric measures (depression, anxiety, and general mental health) (continued)**

|  | **Validation** | **Baseline** | **1 wk/2 wk** | **1 mo** | **6 wk/2 mo** | **3 mo** | **4 mo** | **6 mo** | **9 mo** | **12 mo** | **24 mo** | **60 mo** |
| --- | --- | --- | --- | --- | --- | --- | --- | --- | --- | --- | --- | --- |
|  |  | **Data are shown as "mean [SD] (n)," "N [%] (n)," or “median [IQR] (n)”** | | | | | | | | | | |
| **Psychiatric Status Schedule (PSS: structured interview)** | | | | | | | | | | | | |
| Depression and Anxiety[^34^](#_ENREF_34) | R  V  - |  | E: 10.26 [6.90] (27); C: 10.48 [7.55] (35) at 1 wk |  |  |  | E: 4.89 [5.90] (27); C: 3.00 [2.80] (35) |  |  |  |  |  |
| Suicidal ideation[^34^](#_ENREF_34) | R  V  - |  | E: 0.44 [0.89] (27); C: 0.40 [1.00] (35) at 1 wk |  |  |  | E: 0.22 [0.85] (27); C: 0.04 [0.20] (35) |  |  |  |  |  |
| Social isolation[^34^](#_ENREF_34) | R  V  - |  | E: 2.41 [2.72] (27); C: 1.92 [2.47] (35) at 1 wk |  |  |  | E: 0.93 [2.18] (27); C: 0.04 [0.20] (35) |  |  |  |  |  |
| Social concerns[^34^](#_ENREF_34) | R  V  - |  | E: 0.19 [0.48] (27); C: 0.16 [0.47] (35) at 1 wk |  |  |  | E: 0.37 [1.18] (27); C: 0.08 [0.28] (35) |  |  |  |  |  |
| Daily routine[^34^](#_ENREF_34) | R  V  - |  | E: 0.78 [0.97] (27); C: 0.96 [1.37] (35) at 1 wk |  |  |  | E: 0.33 [0.73] (27); C: 0.32 [0.56] (35) |  |  |  |  |  |
| Total score[^34^](#_ENREF_34) | R  V  - |  | E: 14.06 [7.35] (27); C: 13.29 [19.91] (35) at 1 wk |  |  |  | E: 6.74 [8.14] (27); C: 3.48 [3.22] (35) |  |  |  |  |  |
| General mental health[^35^](#_ENREF_35) | R  V  - |  |  |  |  | E: 19 [83%] (23); C: 28 [85%] (33) ^b^ |  | E: 20 [83%] (24); C: 22 [78%] (28) ^b^ |  |  |  |  |

Abbreviations: wk, week/weeks; mo, month/months; E, experimental intervention group; C, control group.

The Validation column shows whether the psychological measure had been previously shown to have reliability, validity, and predictability. R: Previous evidence for reliability; V: Previous evidence for validity; P: Previous evidence for predictability.

^a^Pre–post difference.

^b^28 and 27 of the 51 participants in E, and 19 and 24 of the 52 participants in C were lost by 3 and 6 months, respectively.[^35^](#_ENREF_35)

See references in Additional file 11.

**Table S7e List of psychometric measures (alcohol-related problems)**

|  | **Validation** | **Baseline** | **1 wk/2 wk** | **1 mo** | **6 wk/2 mo** | **3 mo** | **4 mo** | **6 mo** | **9 mo** | **12 mo** | **24 mo** | **60 mo** |
| --- | --- | --- | --- | --- | --- | --- | --- | --- | --- | --- | --- | --- |
|  |  | **Data are shown as "mean [SD] (n)," "N [%] (n)," or “median [IQR] (n)”** | | | | | | | | | | |
| **Alcohol Use Disorders Identification Test (AUDIT)** | | | | | | | | | | | | |
| Daily total alcohol units[^31^](#_ENREF_31) | R  V  - | E: 5 [0–17.5] (14); C: 3.5 [0–13] (6) |  |  |  | E: 2.5 [0–11.25] (11); C: 0 [0–21] (4) |  |  |  |  |  |  |
| AUDIT total score[^31^](#_ENREF_31)^,^[^35^](#_ENREF_35) | R  V  - | E: 2.5 [0–23] (14); C: 3.5 [0–11.5] (6)[^31^](#_ENREF_31) |  |  |  | E: 3.5 [0–21] (11); C: 0 [0–19.5] (4)[^31^](#_ENREF_31)  E: 20.2 [9.3] (32); C: 21.5 [10.7] (36)[^35^](#_ENREF_35) |  | E: 21.5 [9.3] (29); C: 22.2 [12.4] (33)[^35^](#_ENREF_35) |  |  |  |  |
| **Paddington Alcohol Test (PAT)** | | | | | | | | | | | | |
| Units of alcohol per drinking session[^34^](#_ENREF_34) | -  V  - |  |  |  |  | E: 13.7 [9.9] (28); C: 19.4 [17.6] (31) |  | E: 13.2 [13.1] (28); C: 20.5 [17.7] (31) |  |  |  |  |

Abbreviations: wk, week/weeks; mo, month/months; E, experimental intervention group; C, control group.

The Validation column shows whether the psychological measure had been previously shown to have reliability, validity, and predictability. R: Previous evidence for reliability; V: Previous evidence for validity; P: Previous evidence for predictability.

See references in Additional file 11.

**Table S7f List of psychometric measures (quality of life and global functioning)**

|  | **Validation** | **Baseline** | **1 wk/2 wk** | **1 mo** | **6 wk/2 mo** | **3 mo** | **4 mo** | **6 mo** | **9 mo** | **12 mo** | **24 mo** | **60 mo** |
| --- | --- | --- | --- | --- | --- | --- | --- | --- | --- | --- | --- | --- |
|  |  | **Data are shown as "mean [SD] (n)," "N [%] (n)," or “median [IQR] (n)”** | | | | | | | | | | |
| **EuroQol health measure (EQ-5D) (descriptive score)** | | | | | | | | | | | | |
| Quality of life[^7^](#_ENREF_7) | R  V  - | E: 8.0 [1.9] (319); C: 7.8 [1.8] (349) |  |  |  | E: 7.4 [1.9] (223); C: 7.4 [1.9] (257) |  |  |  | E: 7.4 [1.8] (211); C: 7.1 [1.8] (235) |  |  |
| **Quality of Life Scale** | | | | | | | | | | | | |
| Quality of life[^30^](#_ENREF_30) | -  -  - | E1: 49.28 [12.79] (82); E2: 47.29 [13.87] (80); C: 50.75 [12.34] (77) |  |  |  | E1: 56.60 [16.69] (55); E2: 56.66 [14.54] (56); C: 59.52 [16.71] (61) |  | E1: 67.88 [24.59] (34); E2: 71.14 [22.42] (41); C: 70.72 [21.68] (40) |  | E1: 62.60 [20.55] (24); E2: 61.33 [16.50] (36); C: 63.79 [17.83] (27) |  |  |
| **Global Assessment of Functioning (GAF)** | | | | | | | | | | | | |
| Global functioning[^17^](#_ENREF_17) | R  V  - |  |  | E: 50.8 [19.4] (107); C: 49.7 [20.7] (109)  E: 50.5 [19.9] (no data); C: 50.3 [21.1] (no data) ^a^ |  |  |  |  |  | E: 61.4 [20.4] (no data); C: 58.6 [20.2] (no data) ^a^ |  |  |
| **WHO Well-Being Index** | | | | | | | | | | | | |
| Well-being[^9^](#_ENREF_9) | R  V  - | No data[^9^](#_ENREF_9) |  |  |  |  |  |  |  |  |  |  |

**Table S7f List of psychometric measures (quality of life and global functioning) (continued)**

|  | **Validation** | **Baseline** | **1 wk/2 wk** | **1 mo** | **6 wk/2 mo** | **3 mo** | **4 mo** | **6 mo** | **9 mo** | **12 mo** | **24 mo** | **60 mo** |
| --- | --- | --- | --- | --- | --- | --- | --- | --- | --- | --- | --- | --- |
|  |  | **Data are shown as "mean [SD] (n)," "N [%] (n)," or “median [IQR] (n)”** | | | | | | | | | | |
| **36-Item Short Form Health Survey (SF-36)** | | | | | | | | | | | | |
| Health-related quality of life[^6^](#_ENREF_6) | R  V  - | No data[^6^](#_ENREF_6) |  |  |  |  |  |  |  |  |  |  |
| Physical functioning[^7^](#_ENREF_7) | R  V  - | E: 47.7 [12.2] (311); C: 49.0 [11.2] (347) |  |  |  | E: 47.5 [11.9] (209); C: 48.5 [11.4] (246) |  |  |  | E: 48.8 [11.0] (210); C: 48.6 [10.8] (234) |  |  |
| Mental functioning[^7^](#_ENREF_7) | R  V  - | E: 19.4 [12.8] (311); C: 19.9 [13.8] (347) |  |  |  | E: 31.8 [15.8] (209); C: 28.8 [16.4] (246) |  |  |  | E: 31.0 [16.5] (210); C: 32.9 [17.0] (234) |  |  |
| **Children’s Global Assessment Scale (CGAS)** | | | | | | | | | | | | |
| Severity of psychiatric disturbance[^28^](#_ENREF_28) | R  -  - |  |  | E: 15.6 [6.2] (35); C: 16.0 [6.0] (35) |  |  |  |  |  |  |  |  |
| **Strengths and Difficulties Questionnaire (SDQ)** | | | | | | | | | | | | |
| Strength and difficulties[^28^](#_ENREF_28) | R  V  - |  |  | E: 64.6 [12.9] (35); C: 60.1 [9.9] (35) |  |  |  |  |  |  |  |  |

Abbreviations: wk, week/weeks; mo, month/months; E, experimental intervention group; C, control group.

The Validation column shows whether the psychological measure had been previously shown to have reliability, validity, and predictability. R: Previous evidence for reliability; V: Previous evidence for validity; P: Previous evidence for predictability.

^a^These data were based on 163 (E and C groups) individuals who completed the ratings at both 1 mo and 12 mo.

See references in Additional file 11.

**Table S7g List of psychometric measures (problem solving)**

|  | **Validation** | **Baseline** | **1 wk/2 wk** | **1 mo** | **6 wk/2 mo** | **3 mo** | **4 mo** | **6 mo** | **9 mo** | **12 mo** | **24 mo** | **60 mo** |
| --- | --- | --- | --- | --- | --- | --- | --- | --- | --- | --- | --- | --- |
|  |  | **Data are shown as "mean [SD] (n)," "N [%] (n)," or “median [IQR] (n)”** | | | | | | | | | | |
| **Problem Solving Inventory** | | | | | | | | | | | | |
| Problem solving confidence (F: I)[^24^](#_ENREF_24) | R  V  - | E: 35.9 [7.23] (20); C: 29.9 [5.14] (20) |  |  |  | E: 7.4 [13.46] (20); C: -1.65 [6.41] (20) ^a^ |  |  |  |  |  |  |
| Approach avoidance style (F: II)[^24^](#_ENREF_24) | R  V  - | E: 59.05 [9.05] (20); C: 54.85 [5.80] (20) |  |  |  | E: 11.9 [10.53] (20); C: 2.45 [6.42] (20) ^a^ |  |  |  |  |  |  |
| Personal control (F: III)[^24^](#_ENREF_24) | R  V  - | E: 22.5 [4.38] (20); C: 19.7 [3.93] (20) |  |  |  | E: 5.15 [7.03] (20); C: -1.5 [3.62] (20) ^a^ |  |  |  |  |  |  |
| **Self-Rating Problem-Solving Scale (SRPS; 1987)** | | | | | | | | | | | | |
| Attitude and response to interpersonal problems[^22^](#_ENREF_22) | R  V  - | E: 20.68 [10.50] (19); C: 6.10 [6.92] (20) |  |  |  |  |  |  |  | E: 20.59 [14.07] (17); C: 3.65 [10.55] (16) |  |  |

**Table S7g List of psychometric measures (problem solving) (continued)**

|  | **Validation** | **Baseline** | **1 wk/2 wk** | **1 mo** | **6 wk/2 mo** | **3 mo** | **4 mo** | **6 mo** | **9 mo** | **12 mo** | **24 mo** | **60 mo** |
| --- | --- | --- | --- | --- | --- | --- | --- | --- | --- | --- | --- | --- |
|  |  | **Data are shown as "mean [SD] (n)," "N [%] (n)," or “median [IQR] (n)”** | | | | | | | | | | |
| **Miskimins Self-Goal-Other discrepancy scale (MSGO; 1979)** | | | | | | | | | | | | |
| Self- and ideal concept[^22^](#_ENREF_22) | -  -  - | E: 16.32 [11.13] (19); C: 4.20 [6.24] (20) |  |  |  |  |  |  |  | E: 16.06 [11.69] (17); C: 4.38 [6.24] (16) |  |  |
| **Dysfunctional Attitude Scale (DAS)** | | | | | | | | | | | | |
| Dysfunctional attitude[^24^](#_ENREF_24) | R  V  - | E: 178.15 [22.22] (20); C: 171.30 [14.77] (20) |  |  |  | E: 59.65 [46.98] (20); C: -1.6 [14.36] (20)^a^ |  |  |  |  |  |  |
| **Problem Solving skill (PSI)** | | | | | | | | | | | | |
| Problem-solving skills[^24^](#_ENREF_24) | R  V  - | E: 117.20 [17.69] (20); C: 104.45 [10.68] (20) |  |  |  | E: 24.2 [27.31] (20); C: -0.7 [9.23] (20)^a^ |  |  |  |  |  |  |

**Table S7g List of psychometric measures (problem solving) (continued)**

|  | **Validation** | **Baseline** | **1 wk/2 wk** | **1 mo** | **6 wk/2 mo** | **3 mo** | **4 mo** | **6 mo** | **9 mo** | **12 mo** | **24 mo** | **60 mo** |
| --- | --- | --- | --- | --- | --- | --- | --- | --- | --- | --- | --- | --- |
|  |  | **Data are shown as "mean [SD] (n)," "N [%] (n)," or “median [IQR] (n)”** | | | | | | | | | | |
| **Social Problem-Solving Inventory-revised (SPSI-R)** | | | | | | | | | | | | |
| Positive problem orientation (SPSI-R subscale)[^27^](#_ENREF_27) | R  V  - | E: 4.3 [4.3] (9); C: 5.7 [2.7] (9) |  |  | E: 6.7 [4.2] (9); C: 7.5 [4.0] (9) after intervention |  | E: 11.1 [4.9] (9); C: 7.5 [3.2] (9) 2 mo after intervention |  |  |  |  |  |
| Negative problem orientation (SPSI-R subscale)[^26^](#_ENREF_26)^,^[^27^](#_ENREF_27) | R  V  - | E: 114.85 [no data] (60); C: 115.26 [no data] (60)[^26^](#_ENREF_26)  E: 33.1 [6.1] (9); C: 32.0 [5.2] (9)[^27^](#_ENREF_27) |  |  | E: 24.8 [6.5] (9); C: 31.8 [4.9] (9) after intervention[^27^](#_ENREF_27) |  | E: 20.3 [6.9] (9); C: 32.3 [4.3] (9) 2 mo after intervention[^27^](#_ENREF_27) | E: 102.95 [no data] (60); C: 110.94 [no data] (60)[^26^](#_ENREF_26) |  |  |  |  |
| Rational problem solving (SPSI-R subscale)[^27^](#_ENREF_27) | R  V  - | E: 17.8 [5.9] (9); C: 24.8 [7.3] (9) |  |  | E: 24.8 [6.5] (9); C: 31.8 [4.9] (9) after intervention |  | E: 42.7 [17.0] (9); C: 31.7 [7.2] (9) 2 mo after intervention |  |  |  |  |  |

**Table S7g List of psychometric measures (problem solving) (continued)**

|  | **Validation** | **Baseline** | **1 wk/2 wk** | **1 mo** | **6 wk/2 mo** | **3 mo** | **4 mo** | **6 mo** | **9 mo** | **12 mo** | **24 mo** | **60 mo** |
| --- | --- | --- | --- | --- | --- | --- | --- | --- | --- | --- | --- | --- |
|  |  | **Data are shown as "mean [SD] (n)," "N [%] (n)," or “median [IQR] (n)”** | | | | | | | | | | |
| **Social problem-solving inventory-revised (SPSI-R) (continued)** | | | | | | | | | | | | |
| Impulsivity score (SPSI-R subscale)[^26^](#_ENREF_26)^,^[^27^](#_ENREF_27) | R  V  - | E: 114.69 [no data] (60); C: 110.25 [no data] (60)[^26^](#_ENREF_26)  E: 23.2 [7.6] (9); C: 26.8 [7.1] (9)[^27^](#_ENREF_27) |  |  | E: 20.9 [5.8] (9); C: 25.3 [5.3] (9) after intervention[^27^](#_ENREF_27) |  | E: 15.7 [6.7] (9); C: 26.7 [4.6] (9) 2 mo after intervention[^27^](#_ENREF_27) | E: 103.69 [no data] (60); C: 108.43 [no data] (60)[^26^](#_ENREF_26) |  |  |  |  |
| Avoidance style (SPSI-R subscale)[^27^](#_ENREF_27) | R  V  - | E: 21.2 [5.4] (9); C: 20.7 [4.4] (9) |  |  | E: 18.4 [6.0] (9); C: 20.8 [2.7] (9) after intervention |  | E: 11.8 [5.4] (9); C: 20.2 [2.5] (9) 2 mo after intervention |  |  |  |  |  |

Abbreviations: wk, week/weeks; mo, month/months; E, experimental intervention group; C, control group.

The Validation column shows whether the psychological measure had been previously shown to have reliability, validity, and predictability. R: Previous evidence for reliability; V: Previous evidence for validity; P: Previous evidence for predictability.

^a^Pre–post difference.

See references in Additional file 11.

**Table S7h List of psychometric measures (others)**

|  | **Validation** | **Baseline** | **1 wk/2 wk** | **1 mo** | **6 wk/2 mo** | **3 mo** | **4 mo** | **6 mo** | **9 mo** | **12 mo** | **24 mo** | **60 mo** |
| --- | --- | --- | --- | --- | --- | --- | --- | --- | --- | --- | --- | --- |
|  |  | **Data are shown as "mean [SD] (n)," "N [%] (n)," or “median [IQR] (n)”** | | | | | | | | | | |
| **Social Behavior Assessment Schedule (SBAS)** | | | | | | | | | | | | |
| Patient behavior[^34^](#_ENREF_34) | R  V  - |  | E: 5.06 [4.91] (16); C: 4.55 [3.56] (20) at 1 wk |  |  |  | E: 3.44 [4.32] (16); C: 2.00 [2.70] (20) |  |  |  |  |  |
| Patient social performance[^34^](#_ENREF_34) | R  V  - |  | E: 0.68 [0.46] (16); C: 0.51 [0.40] (20) at 1 wk |  |  |  | E: 0.41 [0.44] (16); C: 3.90 [2.65] (20) |  |  |  |  |  |
| Household burden[^34^](#_ENREF_34) | R  V  - |  | E: 3.06 [1.88] (16); C: 3.90 [2.65] (20) at 1 wk |  |  |  | E: 0.81 [1.05] (16); C: 2.10 [2.99] (20) |  |  |  |  |  |
| Patient behavior –reported distress[^34^](#_ENREF_34) | R  V  - |  | E: 0.47 [0.47] (16); C: 0.55 [0.45] (20) at 1 wk |  |  |  | E: 0.35 [0.43] (16); C: 0.31 [0.44] (20) |  |  |  |  |  |
| Patient social performance –reported distress[^34^](#_ENREF_34) | R  V  - |  | E: 0.27 [0.40] (16); C: 0.30 [0.39] (20) at 1 wk |  |  |  | E: 0.30 [0.39] (16); C: 0.11 [0.20] (20) |  |  |  |  |  |
| Household burden –reported distress[^34^](#_ENREF_34) | R  V  - |  | E: 0.88 [0.44] (16); C: 0.81 [0.46] (20) at 1 wk |  |  |  | E: 0.81 [0.63] (16); C: 0.89 [0.67] (20) |  |  |  |  |  |

**Table S7h List of psychometric measures (others) (continued)**

|  | **Validation** | **Baseline** | **1 wk/2 wk** | **1 mo** | **6 wk/2 mo** | **3 mo** | **4 mo** | **6 mo** | **9 mo** | **12 mo** | **24 mo** | **60 mo** |
| --- | --- | --- | --- | --- | --- | --- | --- | --- | --- | --- | --- | --- |
|  |  | **Data are shown as "mean [SD] (n)," "N [%] (n)," or “median [IQR] (n)”** | | | | | | | | | | |
| **Reinforcement Survey Schedule (RSS)** | | | | | | | | | | | | |
| Reinforcer^[21](#_ENREF_21)^ | -  -  - | E: 215 [115] (12); C: 216 [82] (12) before intervention | E: 210 [106] (12); C: 257 [71] (12) after intervention at day 8  E: 226 [110] (12); C: 243 [71] (12) at 2 wk |  | E: 212 [100] (12); C: 221 [73] (12) at 6 wk | E: 212 [108] (12); C: 242 [87] (12) |  | E: 233 [119] (12); C: 249 [71] (12) | E: 226 [110] (12); C: 259 [99] (12) |  |  |  |
| **Assertiveness Questionnaire (AQ)** | | | | | | | | | | | | |
| Assertiveness[^21^](#_ENREF_21) | -  -  - | E: 12 [4] (12); C: 11 [4] (12) before intervention | E: 16 [6] (12); C: 17 [4] (12) after intervention at day 8  E: 16 [5] (12);  C: 17 [4] (12) at 2 wk |  | E: 17 [4] (12);  C: 17 [5] (12) at 6 wk | E: 16 [5] (12);  C: 19 [5] (12) |  | E: 17 [5] (12);  C: 18 [4] (12) | E: 17 [5] (12);  C: 16 [5] (12) |  |  |  |

**Table S7h List of psychometric measures (others) (continued)**

|  | **Validation** | **Baseline** | **1 wk/2 wk** | **1 mo** | **6 wk/2 mo** | **3 mo** | **4 mo** | **6 mo** | **9 mo** | **12 mo** | **24 mo** | **60 mo** |
| --- | --- | --- | --- | --- | --- | --- | --- | --- | --- | --- | --- | --- |
|  |  | **Data are shown as "mean [SD] (n)," "N [%] (n)," or “median [IQR] (n)”** | | | | | | | | | | |
| **Fear Survey Schedule (FSS)** | | | | | | | | | | | | |
| Fear[^21^](#_ENREF_21) | R  V  - | E: 107 [62] (12); C: 136 [38] (12) before intervention | E: 85 [61] (12);  C: 99 [40] (12) after intervention at day 8  E: 83 [65] (12); C: 103 [43] (12) at 2 wk |  | E: 67 [45] (12); C: 117 [90] (12) at 6 wk | E: 57 [43] (12);  C: 91 [36] (12) |  | E: 60 [38] (12);  C: 80 [29] (12) | E: 65 [42] (12);  C: 97 [59] (12) |  |  |  |
| **Client Satisfaction Questionnaire (CSQ)** | | | | | | | | | | | | |
| Satisfaction with care[^35^](#_ENREF_35) | -  V  - |  |  |  |  | E: 10.0 [-] (30); C: 10.5 [-] (32) |  | E: 11.0 [-] (28); C: 9.0 [-] (28) |  |  |  |  |
| **Eysenck Impulsiveness Scale** | | | | | | | | | | | | |
| Impulsiveness^8^ | R, V ,- | No data^8^ |  |  |  |  |  |  |  |  |  |  |
| **Spielberger Trait-Anger Scale** | | | | | | | | | | | | |
| Anger^8^ | R ,V ,- | No data^8^ |  |  |  |  |  |  |  |  |  |  |
| **Bille-Brahe Measurement of Social Support** | | | | | | | | | | | | |
| Social support^8^ | - ,- ,- | No data^8^ |  |  |  |  |  |  |  |  |  |  |

Abbreviations: wk, week/weeks; mo, month/months; E, experimental intervention group; C, control group.

The Validation column shows whether the psychological measure had been previously shown to have reliability, validity, and predictability. R: Previous evidence for reliability; V: Previous evidence for validity; P: Previous evidence for predictability.

See references in Additional file 11.
