# Supplementary material for: Active contact and follow-up interventions to prevent repeat suicide attempts during high-risk periods among patients admitted to emergency departments for suicidal behavior: a systematic review and meta-analysis
Source: BMC Psychiatry. 2019 Jan 25;19:44. doi: 10.1186/s12888-019-2017-7 (PMC6347824; doi:10.1186/s12888-019-2017-7)
Supplement: Supplementary file 9 — List of other outcomes. (DOCX 244 kb) [file 12888_2019_2017_MOESM9_ESM.docx]

**Table S8 List of other outcomes**

|  | **Baseline** | **1 wk/2 wk** | **1 mo** | **6 wk/2 mo** | **3 mo** | **4 mo** | **6 mo** | **9 mo** | **12 mo** | **24 mo** | **60 mo** |
| --- | --- | --- | --- | --- | --- | --- | --- | --- | --- | --- | --- |
|  | **Data are shown as "mean [SD] (n)," "N [%] (n)," or “median [IQR] (n)”** | | | | | | | | | | |
| **Resource utilization: admission to general hospital or any hospital** | | | | | | | | | | | |
| Number of patients admitted to general hospitals for a reason other than self-harm[^7^](#_ENREF_7) |  |  |  |  |  |  |  |  | E: 126 [40%] (318); C: 130 [37%] (351) |  |  |
| Total number of admissions to general hospitals[^7^](#_ENREF_7) |  |  |  |  |  |  |  |  | E: 301 [-] (-); C: 350 [-] (-) |  |  |
| Mean number of days spent in hospital[^4^](#_ENREF_4)^,^[^18^](#_ENREF_18) |  |  |  |  |  |  |  |  | E: 33 [73.5] (140); C: 37 [83.0] (134) (including experimental ward)[^4^](#_ENREF_4)  E1: 21 [13] (107); E2: 27 [19] (95); C: 24 [32] (280)[^18^](#_ENREF_18) |  |  |

**Table S8 List of other outcomes (continued)**

|  | **Baseline** | **1 wk/2 wk** | **1 mo** | **6 wk/2 mo** | **3 mo** | **4 mo** | **6 mo** | **9 mo** | **12 mo** | **24 mo** | **60 mo** |  |
| --- | --- | --- | --- | --- | --- | --- | --- | --- | --- | --- | --- | --- |
|  | **Data are shown as "mean [SD] (n)," "N [%] (n)," or “median [IQR] (n)”** | | | | | | | | | | |  |
| **Resource utilization: admission to general hospital or any hospital (continued)** | | | | | | | | | | | | |
| Mean number of contacts with inpatient treatment[^18^](#_ENREF_18)^,^[^23^](#_ENREF_23) |  |  |  |  |  |  | E: 0.2 [0.62] (46); C: 0.35 [1.7] (48)[^23^](#_ENREF_23) |  | E1: 0.3 [0.6] (107); E2: 0.29 [0.7] (95); C: 0.39 [1] (280)[^18^](#_ENREF_18) |  |  |  |
| Mean number of contacts with ED[^19^](#_ENREF_19)^,^[^23^](#_ENREF_23) |  |  |  |  |  |  | E: 0.52 [0.78] (46); C: 0.4 [0.76] (48) |  |  |  |  |  |
| Median number of contacts with ED[^19^](#_ENREF_19)^,^[^23^](#_ENREF_23) |  |  |  |  |  |  | E: 0 [0–3] (46); C: 0 [0–3] (48)[^23^](#_ENREF_23) |  | E: 1 [0–3] (33); C: 1 [0–2] (33)[^19^](#_ENREF_19) |  |  |  |
| Median number of days spent in hospital[^19^](#_ENREF_19) |  |  |  |  |  |  |  |  | E: 0 [0–1] (33); C: 0 [0–1] (33)[^19^](#_ENREF_19) |  |  |  |

**Table S8 List of other outcomes (continued)**

|  | **Baseline** | **1 wk/2 wk** | **1 mo** | **6 wk/2 mo** | **3 mo** | **4 mo** | **6 mo** | **9 mo** | **12 mo** | **24 mo** | **60 mo** |  |
| --- | --- | --- | --- | --- | --- | --- | --- | --- | --- | --- | --- | --- |
|  | **Data are shown as "mean [SD] (n)," "N [%] (n)," or “median [IQR] (n)”** | | | | | | | | | | |  |
| **Resource utilization: admission to psychiatric hospital** | | | | | | | | | | | | |
| Number of patients admitted to any hospital ward[^18^](#_ENREF_18) |  |  |  |  |  |  |  |  | E1: 21 [22%] (107); E2: 21 [24%] (95); C: 62 [22%] (280)[^18^](#_ENREF_18) |  |  |  |
| Number of patients admitted to psychiatric wards[^4^](#_ENREF_4)^,^[^7^](#_ENREF_7)^,^[^13^](#_ENREF_13)^,^[^19^](#_ENREF_19)^,^[^34^](#_ENREF_34) |  | E: 1 [4%] (27); C: 2 [6%] (35) at 1 wk^[34](#_ENREF_34)^ |  |  |  | E: 3 [11%] (27); C: 4 [11%] (35)[^34^](#_ENREF_34) |  |  | E: 32 [23%] (140); C: 44 [33%] (134)[^4^](#_ENREF_4)  E: 122 [87%] (140); C: 44 [33%] (134) (including experimental ward)[^4^](#_ENREF_4)  E: 32 [10%] (318); C: 36 [10%] (351)[^7^](#_ENREF_7)  E: 1 [3%] (32); C: 2 [6%] (32)[^19^](#_ENREF_19) |  | E: 144 [38%] (378); C: 140 [36%] (394)[^13^](#_ENREF_13) |  |

**Table S8 List of other outcomes (continued)**

|  | **Baseline** | **1 wk/2 wk** | **1 mo** | **6 wk/2 mo** | **3 mo** | **4 mo** | **6 mo** | **9 mo** | **12 mo** | **24 mo** | **60 mo** |  |
| --- | --- | --- | --- | --- | --- | --- | --- | --- | --- | --- | --- | --- |
|  | **Data are shown as "mean [SD] (n)," "N [%] (n)," or “median [IQR] (n)”** | | | | | | | | | | |  |
| **Resource utilization: admission to psychiatric hospital (continued)** | | | | | | | | | | | | |
| Total number of admissions to psychiatric hospitals[^7^](#_ENREF_7)^,^[^13^](#_ENREF_13) |  |  |  |  |  |  |  |  | E: 42 [-] (-); C: 51 [-] (-)[^7^](#_ENREF_7) |  | E: 447 [-] (378); C: 710 [-] (394)[^13^](#_ENREF_13) |  |
| Mean number of days spent in psychiatric hospital[^4^](#_ENREF_4) |  |  |  |  |  |  |  |  | E: 36 [76.06] (140); C: 106 [111.2] (134) |  |  |  |
| **Resource utilization: outpatient treatment** | | | | | | | | | | | | |
| Number of patients who received outpatient treatment[^4^](#_ENREF_4) |  |  |  |  |  |  |  |  | E: 119 [85%] (140); C: 64 [48%] (134) |  |  |  |
| Number of patients with no contact with any outpatient services[^15^](#_ENREF_15)^,^[^16^](#_ENREF_16) |  |  |  |  |  |  |  |  | E: 750 [72%] (1043); C: 779 [73%] (1070)[^15^](#_ENREF_15) | E: 537 [54%] (997); C: 573 [57%] (1004)[^16^](#_ENREF_16) |  |  |
| Mean number of contacts with outpatient treatment services[^4^](#_ENREF_4)^,^[^23^](#_ENREF_23) |  |  |  |  |  |  |  |  | E: 14.3 [24.2] (140); C: 11.4 [27.7] (134)[^4^](#_ENREF_4)  E: 1.0 [2.6] (46); C: 0.5 [1.3] (48)[^23^](#_ENREF_23) |  |  |  |

**Table S8 List of other outcomes (continued)**

|  | **Baseline** | **1 wk/2 wk** | **1 mo** | **6 wk/2 mo** | **3 mo** | **4 mo** | **6 mo** | **9 mo** | **12 mo** | **24 mo** | **60 mo** |  |
| --- | --- | --- | --- | --- | --- | --- | --- | --- | --- | --- | --- | --- |
|  | **Data are shown as "mean [SD] (n)," "N [%] (n)," or “median [IQR] (n)”** | | | | | | | | | | |  |
| **Resource utilization: GP treatment (continued)** | | | | | | | | | | | | |
| Number of patients with contact with GPs[^7^](#_ENREF_7)^,^[^15^](#_ENREF_15)^,^[^16^](#_ENREF_16) |  |  |  |  | E: 171 [75%] (227); C: 181 [70%] (258) (consulted a GP)[^7^](#_ENREF_7) |  |  |  | E: 192 [91%] (211); C: 207 [89%] (232) (consulted a GP)[^7^](#_ENREF_7)  E: 2 [0.2%] (1043); C: 4 [0.4%] (1070)[^15^](#_ENREF_15) | E: 7 [1%] (997); C: 4 [0.4%] (1004)[^16^](#_ENREF_16) |  |  |
| Number of patients who talked to GPs about suicide[^18^](#_ENREF_18) |  |  |  |  |  |  |  |  | E1: 82 [77%] (107); E2: 72 [76%] (95); C: 204 [73%] (280) |  |  |  |
| Mean number of contacts with GPs[^7^](#_ENREF_7)^,^[^23^](#_ENREF_23) |  |  |  |  | E: 2.5 [2.2] (327); C: 2.9 [3.3] (357)[^7^](#_ENREF_7) |  | E: 4.7 [5.9] (46); C: 4.7 [5.9] (48)[^23^](#_ENREF_23) |  | E: 4.9 [5.6] (327); C: 5.1 [11.3] (357)[^7^](#_ENREF_7) |  |  |  |

**Table S8 List of other outcomes (continued)**

|  | **Baseline** | **1 wk/2 wk** | **1 mo** | **6 wk/2 mo** | **3 mo** | **4 mo** | **6 mo** | **9 mo** | **12 mo** | **24 mo** | **60 mo** |  |
| --- | --- | --- | --- | --- | --- | --- | --- | --- | --- | --- | --- | --- |
|  | **Data are shown as "mean [SD] (n)," "N [%] (n)," or “median [IQR] (n)”** | | | | | | | | | | |  |
| **Resource utilization: mental health services or other professionals** | | | | | | | | | | | | |
| Number of patients with contact with alcohol and drug services[^7^](#_ENREF_7) |  |  |  |  | E: 34 [11%] (320); C: 58 [16%] (354) (face-to-face contact) |  |  |  | E: 32 [10%] (318); C: 48 [14%] (351) (face-to-face contact) |  |  |  |
| Number of patients with no recorded face-to-face contact with mental health services[^7^](#_ENREF_7) |  |  |  |  | E: 125 [39%] (320); C: 165 [47%] (354) |  |  |  | E: 194 [61%] (318); C: 217 [62%] (351) |  |  |  |
| Number of patients with contact with any professionals[^16^](#_ENREF_16) |  |  |  |  |  |  |  |  |  | E: 460 [46%] (997); C: 431 [43%] (1004) |  |  |
| Number of patients with contact with physicians[^15^](#_ENREF_15)^,^[^16^](#_ENREF_16) |  |  |  |  |  |  |  |  | E: 65 [6%] (1043); C: 61 [6%] (1070)[^15^](#_ENREF_15) | E: 138 [14%] (997); C: 121 [12%] (1004)[^16^](#_ENREF_16) |  |  |
| Number of patients with contact with neurologists[^15^](#_ENREF_15)^,^[^16^](#_ENREF_16) |  |  |  |  |  |  |  |  | E: 27 [3%] (1043); C: 27 [3%] (1070)[^15^](#_ENREF_15) | E: 33 [3%] (997); C: 29 [3%] (1004)[^16^](#_ENREF_16) |  |  |

**Table S8 List of other outcomes (continued)**

|  | **Baseline** | **1 wk/2 wk** | **1 mo** | **6 wk/2 mo** | **3 mo** | **4 mo** | **6 mo** | **9 mo** | **12 mo** | **24 mo** | **60 mo** |  |
| --- | --- | --- | --- | --- | --- | --- | --- | --- | --- | --- | --- | --- |
|  | **Data are shown as "mean [SD] (n)," "N [%] (n)," or “median [IQR] (n)”** | | | | | | | | | | |  |
| **Resource utilization: mental health services or other professionals (continued)** | | | | | | | | | | | | |
| Number of patients with contact with cardiologists[^15^](#_ENREF_15)^,^[^16^](#_ENREF_16) |  |  |  |  |  |  |  |  | E: 11 [1%] (1043); C: 14 [1%] (1070)[^15^](#_ENREF_15) | E: 25 [3%] (997); C: 32 [3%] (1004)[^16^](#_ENREF_16) |  |  |
| Number of patients with contact with gastroenterologists[^16^](#_ENREF_16) |  |  |  |  |  |  |  |  |  | E: 27 [3%] (997); C: 14 [1%] (1004) |  |  |
| Number of patients with contact with endocrinologists[^16^](#_ENREF_16) |  |  |  |  |  |  |  |  |  | E: 12 [1%] (997); C: 10 [1%] (1004) |  |  |
| Number of patients with contact with psychologists[^15^](#_ENREF_15)^,^[^16^](#_ENREF_16) |  |  |  |  |  |  |  |  | E: 4 [0.4%] (1043); C: 1 [0.1%] (1070)[^15^](#_ENREF_15) | E: 27 [3%] (997); C: 15 [1%] (1004)[^16^](#_ENREF_16) |  |  |

**Table S8 List of other outcomes (continued)**

|  | **Baseline** | **1 wk/2 wk** | **1 mo** | **6 wk/2 mo** | **3 mo** | **4 mo** | **6 mo** | **9 mo** | **12 mo** | **24 mo** | **60 mo** |  |
| --- | --- | --- | --- | --- | --- | --- | --- | --- | --- | --- | --- | --- |
|  | **Data are shown as "mean [SD] (n)," "N [%] (n)," or “median [IQR] (n)”** | | | | | | | | | | |  |
| **Resource utilization: mental health services or other professionals (continued)** | | | | | | | | | | | | |
| Number of patients with contact with other medical specialists[^16^](#_ENREF_16) |  |  |  |  |  |  |  |  |  | E: 19 [2%] (997); C: 19 [2%] (1004) |  |  |
| Number of patients with contact with psychiatric/mental health services[^15^](#_ENREF_15)^,^[^16^](#_ENREF_16)^,^[^20^](#_ENREF_20) |  |  |  |  |  |  |  |  | E: 99 [9%] (1043); C: 91 [9%] (1070)[^15^](#_ENREF_15)  E: 44 [22%] (200); C: 63 [32%] (200)[^20^](#_ENREF_20) | E: 243 [24%] (997); C: 258 [26%] (1004)[^16^](#_ENREF_16) |  |  |
| Number of patients with contact with social service departments[^20^](#_ENREF_20) |  |  |  |  |  |  |  |  | E: 34 [17%] (200); C: 47 [24%] (200) |  |  |  |
| Total number of days treatment in mental health services[^29^](#_ENREF_29) |  |  |  |  |  |  |  |  |  | E: 303.2 [286.6] (35); C: 243.7 [265.6] (34) |  |  |

**Table S8 List of other outcomes (continued)**

|  | **Baseline** | **1 wk/2 wk** | **1 mo** | **6 wk/2 mo** | **3 mo** | **4 mo** | **6 mo** | **9 mo** | **12 mo** | **24 mo** | **60 mo** |  |
| --- | --- | --- | --- | --- | --- | --- | --- | --- | --- | --- | --- | --- |
|  | **Data are shown as "mean [SD] (n)," "N [%] (n)," or “median [IQR] (n)”** | | | | | | | | | | |  |
| **Resource utilization : mental health services or other professionals (continued)** | | | | | | | | | | | | |
| Mean number of contacts with mental health services[^7^](#_ENREF_7) |  |  |  |  | E: 4.3 [7.6] (320); C: 3.0 [5.2] (354) (face-to-face contact) |  |  |  | E: 5.4 [11.6] (318);C: 4.9 [10.5] (351) (face-to-face contact) |  |  |  |
| Mean number of contacts with other mental health professionals[^23^](#_ENREF_23) |  |  |  |  |  |  | E: 4.0 [11.5] (46); C: 3.1 [8.1] (48) |  |  |  |  |  |
| Median number of patients with contact with psychiatric/mental health services[^19^](#_ENREF_19)^,^[^20^](#_ENREF_20) |  |  |  |  |  |  |  |  | E: 2 [0–9] (33); C: 0 [0–2] (33)[^18^](#_ENREF_18)^,^ |  |  |  |

**Table S8 List of other outcomes (continued)**

|  | **Baseline** | **1 wk/2 wk** | **1 mo** | **6 wk/2 mo** | **3 mo** | **4 mo** | **6 mo** | **9 mo** | **12 mo** | **24 mo** | **60 mo** |  |
| --- | --- | --- | --- | --- | --- | --- | --- | --- | --- | --- | --- | --- |
|  | **Data are shown as "mean [SD] (n)," "N [%] (n)," or “median [IQR] (n)”** | | | | | | | | | | |  |
| **Received psychiatric treatment** | | | | | | | | | | | | |
| Number of patients taking any psychiatric medication[^7^](#_ENREF_7)^,^[^18^](#_ENREF_18)^,^[^30^](#_ENREF_30) |  |  |  |  | E: 146 [65%] (226); C: 111 [53%] (211)[^7^](#_ENREF_7)  E1: 6 [7.3% in 82] (55 were assessed); E2: 10 [12.5% in 80] (56 were assessed); C: 9 [11.7% in 77] (61 were assessed)[^30^](#_ENREF_30) |  | E1: 3 [3.7% in 82] (34 were assessed); E2: 7 [8.8% in 80] (56 were assessed); C: 5 [6.5% in 77] (40 were assessed)[^30^](#_ENREF_30) |  | E: 164 [64%] (256); C: 132 [57%] (232)[^7^](#_ENREF_7)  E1: 43 [%] (107); E2: 30 [%] (95); C: 115 [%] (280)[^18^](#_ENREF_18)  E1: 3 [3.7% in 82] (24 were assessed); E2: 4 [5.0% in 80] (36 were assessed); C: 4 [5.2% in 77] (27 were assessed)[^30^](#_ENREF_30) |  |  |  |

**Table S8 List of other outcomes (continued)**

|  | **Baseline** | **1 wk/2 wk** | **1 mo** | **6 wk/2 mo** | **3 mo** | **4 mo** | **6 mo** | **9 mo** | **12 mo** | **24 mo** | **60 mo** |  |
| --- | --- | --- | --- | --- | --- | --- | --- | --- | --- | --- | --- | --- |
|  | **Data are shown as "mean [SD] (n)," "N [%] (n)," or “median [IQR] (n)”** | | | | | | | | | | |  |
| **Received psychiatric treatment (continued)** | | | | | | | | | | | | |
| Number of patients who received anxiolytics[^18^](#_ENREF_18) |  |  |  |  |  |  |  |  | E1: 15 [%] (107); E2: 15 [%] (95); C: 16 [%] (280) |  |  |  |
| Number of patients who received antidepressants[^18^](#_ENREF_18) |  |  |  |  |  |  |  |  | E1: 26 [%] (107); E2: 20 [%] (95); C: 19 [%] (280) |  |  |  |
| Number of patients who received psychotherapies[^18^](#_ENREF_18) |  |  |  |  |  |  |  |  | E1: 42 [%] (107); E2: 31 [%] (95); C: 104 [%] (280) in 13 mo |  |  |  |

**Table S8 List of other outcomes (continued)**

|  | **Baseline** | **1 wk/2 wk** | **1 mo** | **6 wk/2 mo** | **3 mo** | **4 mo** | **6 mo** | **9 mo** | **12 mo** | **24 mo** | **60 mo** |  |
| --- | --- | --- | --- | --- | --- | --- | --- | --- | --- | --- | --- | --- |
|  | **Data are shown as "mean [SD] (n)," "N [%] (n), or “median [IQR] (n)”** | | | | | | | | | | |  |
| **Adherence to referral after intervention** | | | | | | | | | | | | |
| Number of patients who attended first sessions[^28^](#_ENREF_28) |  |  |  |  | E: 29 [83%] (35); C: 17 [49%] (35) during 3 mo follow-up period |  |  |  |  |  |  |  |
| Number of patients who attended 4 or more sessions[^28^](#_ENREF_28) |  |  |  |  | E: 14 [40%] (35); C: 4 [11%] (35) during 3 mo follow-up period |  |  |  |  |  |  |  |
| Number of patients who attended at least 1 follow-up appointment[^28^](#_ENREF_28) |  |  |  |  | E: 30 [86%] (35); C: 18 [51%] (35) during 3 mo follow-up period |  |  |  |  |  |  |  |

**Table S8 List of other outcomes (continued)**

|  | **Baseline** | **1 wk/2 wk** | **1 mo** | **6 wk/2 mo** | **3 mo** | **4 mo** | **6 mo** | **9 mo** | **12 mo** | **24 mo** | **60 mo** |  |
| --- | --- | --- | --- | --- | --- | --- | --- | --- | --- | --- | --- | --- |
|  | **Data are shown as "mean [SD] (n)," "N [%] (n)," or “median [IQR] (n)”** | | | | | | | | | | |  |
| **Employment status** | | | | | | | | | | | | |
| Number of patients with time off work[^34^](#_ENREF_34) |  | E: 4 [15%] (27); C: 5 [14%] (35) at 1 wk |  |  |  |  |  |  |  |  |  |  |
| Number of patients with employment at 1 wk but unemployed at 16 wk^[34](#_ENREF_34)^ |  |  |  |  |  | E: 3 [11%] (27); C: 5 [14%] (35) |  |  |  |  |  |  |
| Number of patients unemployed at 1 wk, but in employment at 16 wk^[34](#_ENREF_34)^ |  |  |  |  |  | E: 2 [7%] (27); C: 2 [6%] (35) |  |  |  |  |  |  |

**Table S8 List of other outcomes (continued)**

|  | **Baseline** | **1 wk/2 wk** | **1 mo** | **6 wk/2 mo** | **3 mo** | **4 mo** | **6 mo** | **9 mo** | **12 mo** | **24 mo** | **60 mo** |  |
| --- | --- | --- | --- | --- | --- | --- | --- | --- | --- | --- | --- | --- |
|  | **Data are shown as "mean [SD] (n)," "N [%] (n)," or “median [IQR] (n)”** | | | | | | | | | | |  |
| **Others** | | | | | | | | | | | | |
| Number of patients on probation[^20^](#_ENREF_20) |  |  |  |  |  |  |  |  | E: 13 [7%] (200); C: 10 [5%] (200) |  |  |  |
| Number of patients with improvement in social problems assessed by a semi-structured questionnaire[^20^](#_ENREF_20) |  |  |  |  |  | E: 70 [86%] (81); C: 53 [70%] (76)^a^ |  |  |  |  |  |  |
| Number of patients with suicide ideation (number of participants responding “Yes”)[^15^](#_ENREF_15)^,^[^16^](#_ENREF_16)^,^[^21^](#_ENREF_21) | E: 12 [100%] (12); C: 12 [100] (12) before intervention[^21^](#_ENREF_21) | E: 5 [42] (12); C: 7 [58] (12) at 2 wk^[21](#_ENREF_21)^ |  | E: 3 [25] (12); C: 7 [58] (12) at 6 wk^[21](#_ENREF_21)^ | E: 6 [50] (12); C: 6 [50] (12)[^21^](#_ENREF_21) |  | E: 3 [25] (12); C: 8 [67] (12)[^21^](#_ENREF_21) | E: 3 [25] (12); C: 9 [75] (12)[^21^](#_ENREF_21) | E: 302 [29%] (1043); C: 446 [42%] (1070)[^15^](#_ENREF_15)  E: 5 [42] (12); C: 9 [75] (12)[^21^](#_ENREF_21) | E: 465 [47%] (997); C: 588 [59%] (1004)[^16^](#_ENREF_16) |  |  |
| Median number of non-suicidal self-harm episodes[^31^](#_ENREF_31) | E: 1 [0–9] (14); C: 9 [0.75–35.25] (6) |  |  |  | E: 0 [0–4.25] (11); C: 6 [1–23] (4) |  |  |  |  |  |  |  |

**Table S8 List of other outcomes (continued)**

|  | **Baseline** | **1 wk/2 wk** | **1 mo** | **6 wk/2 mo** | **3 mo** | **4 mo** | **6 mo** | **9 mo** | **12 mo** | **24 mo** | **60 mo** |
| --- | --- | --- | --- | --- | --- | --- | --- | --- | --- | --- | --- |
|  | **Data are shown as "mean [SD] (n)," "N [%] (n)," or “median [IQR] (n)”** | | | | | | | | | | |
| **Others** | | | | | | | | | | | |
| Number of patients with urgent suicide ideation (number of participants responding “Yes”)[^21^](#_ENREF_21) | E: 9 [75%] (12); C: 4 [33%] (12) before intervention | E: 0 [0%] (12); C: 2 [17%] (12) at 2 wk |  | E: 3 [25%] (12); C: 4 [33%] (12) at 6 wk | E: 2 [17%] (12);  C: 4 [33%] (12) |  | E: 0 [0%] (12); C: 5 [42%] (12) | E: 1 [8%] (12); C: 4 [33%] (12) | E: 1 [8%] (12); C: 4 [33%] (12) |  |  |
| Number of patients with suicide plans (number of participants responding “Yes”)[^21^](#_ENREF_21) | E: 7 [58%] (12); C: 7 [58%] (12) before intervention |  | E: 1 [8%] (12); C: 4 [33%] (12) at 2 wk | E: 1 [8%] (12); C: 4 [33%] (12) at 6 wk | E: 1 [8%] (12); C: 4 [33%] |  | E: 1 [8%] (12); C: 5 [42%] (12) | E: 1 [8%] (12); C: 5 [42%] (12) | E: 0 [0%] (12); C: 4 [33%] (12) |  |  |
| Number of patients with self-cutting (mutilation) (number of participants responding “Yes”)[^15^](#_ENREF_15)^,^[^16^](#_ENREF_16) |  |  |  |  |  |  |  |  | E: 42 [4%] (1043); C: 50 [5%] (1070)[^15^](#_ENREF_15) | E: 15 [2%] (997); C: 15 [1%] (1004)[^16^](#_ENREF_16) |  |
| Number of patients with subsequent self-harm that involved use of alcohol[^35^](#_ENREF_35) |  |  |  |  | E: 7 [88%] (8); C: 10 [63%] (16) |  | E: 6 [67%] (9); C: 8 [73%] (11) |  |  |  |  |
| Mean of satisfaction with treatment (10-point scale from 0 = not satisfied to 10 = extremely satisfied)[^23^](#_ENREF_23) |  |  | E: 6.56 [3.42] (46); C: 4.40 [3.08] (43) after 1-mo intervention |  |  |  | E: 5.46 [3.38] (47); C: 3.89 [2.76] 48) |  |  |  |  |

**Table S8 List of other outcomes (continued)**

|  | **Baseline** | **1 wk/2 wk** | **1 mo** | **6 wk/2 mo** | **3 mo** | **4 mo** | **6 mo** | **9 mo** | **12 mo** | **24 mo** | **60 mo** |
| --- | --- | --- | --- | --- | --- | --- | --- | --- | --- | --- | --- |
|  | **Data are shown as "mean [SD] (n)," "N [%] (n)," or “median [IQR] (n)”** | | | | | | | | | | |
| **Others** | | | | | | | | | | | |
| Mean of hostility on a checklist item created by the authors (higher scores indicate stronger hostility)[^24^](#_ENREF_24) | E: 38.1 [9.67] (20); C: 37.1 [7.43] (20) |  |  |  | E: 8.45 [11.1] (20); C: 0.4 [7.08] (20)^b^ |  |  |  |  |  |  |
| Mean of impulsivity on a checklist item created by the authors (higher scores indicate severer impulsivity)[^24^](#_ENREF_24) | E: 39.35 [11.51] (20); C: 38.20 [6.46] (20) |  |  |  | E: 7.45 [12.59] (20); C: 1.05 [7.1] (20)^b^ |  |  |  |  |  |  |
| Types and numbers of people or organizations consulted^5^ | No data^5^ |  |  |  |  |  |  |  |  |  |  |
| Medical services utilization (clinical visit or hospital admission)^5^ | No data^5^ |  |  |  |  |  |  |  |  |  |  |
| Physical function^5^ | No data^5^ |  |  |  |  |  |  |  |  |  |  |

Abbreviations: wk, week/weeks; mo, month/months; E, experimental intervention group; C, control group; ED, emergency department.

^a^The authors examined the reliability in a pilot study.

^b^Pre–post difference.

See references in Additional file 11.
