# Supplementary material for: Active contact and follow-up interventions to prevent repeat suicide attempts during high-risk periods among patients admitted to emergency departments for suicidal behavior: a systematic review and meta-analysis
Source: BMC Psychiatry. 2019 Jan 25;19:44. doi: 10.1186/s12888-019-2017-7 (PMC6347824; doi:10.1186/s12888-019-2017-7)
Supplement: Supplementary file 10 — Risk of bias. (DOCX 82 kb) [file 12888_2019_2017_MOESM10_ESM.docx]

**Table S9 Risk of bias**

|  | **Random sequence generation** | **Allocation sequence concealment** | **Blinding of participants and personnel** | **Blinding of outcome assessment** | **Incomplete outcome data** | **Selective outcome reporting** | **Other potential sources of bias** |
| --- | --- | --- | --- | --- | --- | --- | --- |
| **Active contact and follow-up group (Intensive care plus outreach)** | | | | | | | |
| Allard et al., 1992[^2^](#_ENREF_2) | Unclear | Low | Unclear | Unclear | High | High | High |
| Van Heeringen et al., 1995[^3^](#_ENREF_3) | Low | Unclear | Unclear | Unclear | High | High | High |
| van der Sande et al., 1997[^4^](#_ENREF_4) | Low | Low | Unclear | High | Unclear | Low | High |
| Morthorst et al., 2012[^5^](#_ENREF_5) | Low | Low | Unclear | High | Low | High | Low |
| Kawanishi et al., 2014[^6^](#_ENREF_6) | Low | Low | Unclear | Low | Low | Low | Low |
| Hatcher et al., 2015[^7^](#_ENREF_7) | Low | Low | Unclear | Unclear | Low | Low | Unclear |
| **Active contact and follow-up group (Brief intervention and contact)** | | | | | | | |
| Fleischmann et al., 2008[^8^](#_ENREF_8);  Bertolote et al., 2010[^9^](#_ENREF_9) | Low | Low | Unclear | Unclear | High | Low | High |
| Mousavi et al., 2014[^10^](#_ENREF_10) | Unclear | Unclear | Unclear | Unclear | Unclear | High | Unclear |
| **Active contact and follow-up group (Letter or postcard)** | | | | | | | |
| Carter et al., 2005[^11^](#_ENREF_11); 2007[^12^](#_ENREF_12); 2013[^13^](#_ENREF_13) | Low | Low | Unclear | Low | Unclear | High | High |
| Beautrais et al., 2010[^14^](#_ENREF_14) | Low | Low | Unclear | Low | Low | High | High |
| Hassanian-Moghaddam et al., 2011[^15^](#_ENREF_15); 2015[^16^](#_ENREF_16) | Low | Low | Unclear | High | Low | Low | Low |
| **Active contact and follow-up group (Telephone)** | | | | | | | |
| Cedereke et al., 2002[^17^](#_ENREF_17) | Low | Low | Unclear | Low | Low | High | High |
| Vaiva et al., 2006[^18^](#_ENREF_18) | Low | Low | Unclear | Low | High | High | High |
| **Active contact and follow-up group (Composite of letter/postcard and telephone)** | | | | | | | |
| Kapur et al., 2013[^19^](#_ENREF_19) | Low | Low | Unclear | Low | Low | High | High |

**Table S9 Risk of bias (continued)**

|  | **Random sequence generation** | **Allocation sequence concealment** | **Blinding of participants and personnel** | **Blinding of outcome assessment** | **Incomplete outcome data** | **Selective outcome reporting** | **Other potential sources of bias** |
| --- | --- | --- | --- | --- | --- | --- | --- |
| **Psychotherapy group** | | | | | | | |
| Gibbons et al., 1978[^20^](#_ENREF_20) | High | High | Unclear | Low | High | High | Unclear |
| Liberman et al., 1981[^21^](#_ENREF_21) | Unclear | Unclear | Unclear | Unclear | Low | High | High |
| McLeavey et al., 1994[^22^](#_ENREF_22) | Unclear | Unclear | Unclear | Low | Unclear | High | High |
| Guthrie et al., 2001[^23^](#_ENREF_23) | Low | Unclear | Unclear | Low | Low | Low | High |
| Raj et al., 2001[^24^](#_ENREF_24) | High | High | Unclear | Unclear | Unclear | High | High |
| Brown et al., 2005[^25^](#_ENREF_25); Ghahramanlou-Holloway et al, 2012[^26^](#_ENREF_26) | Low | Low | High | High | Low | Low | High |
| Bannan, 2010[^27^](#_ENREF_27) | Low | Low | High | Low | Unclear | High | High |
| Ougrin et al., 2011[^28^](#_ENREF_28), 2013[^29^](#_ENREF_29) | Low | Low | High | Unclear | High | Low | High |
| Wei et al., 2013[^30^](#_ENREF_30) | Low | Unclear | Unclear | Unclear | High | High | High |
| Davidson et al., 2014[^31^](#_ENREF_31) | Low | Unclear | Unclear | Low | High | High | Unclear |
| **Pharmacotherapy group** | | | | | | | |
| Battaglia et al., 1999[^32^](#_ENREF_32) | Unclear | Unclear | High | Unclear | High | High | High |
| **Miscellaneous group** | | | | | | | |
| Torhorst et al., 1987[^33^](#_ENREF_33) | Unclear | Unclear | Unclear | Unclear | High | High | High |
| Waterhouse et al., 1990[^34^](#_ENREF_34) | Unclear | Low | Unclear | High | Low | High | Unclear |
| Crawford et al., 2010[^35^](#_ENREF_35) | Low | Low | Unclear | Unclear | Low | Low | High |

Abbreviations: Low, low risk of bias (plausible bias unlikely to seriously alter the results); Unclear, unclear risk of bias (plausible bias that raises some doubts about the results); High, high risk of bias (plausible bias that seriously weakens confidence in the results).

We referred to and modified data from a previous paper (Inagaki M, Kawashima Y, Kawanishi C, et al. Interventions to prevent repeat suicidal behavior in patients admitted to an emergency department for a suicide attempt: a meta-analysis. *J Affect Disord* 2015; **175**: 66-78).

See references in Additional file 11.
