# Supplementary material for: Active contact and follow-up interventions to prevent repeat suicide attempts during high-risk periods among patients admitted to emergency departments for suicidal behavior: a systematic review and meta-analysis
Source: BMC Psychiatry. 2019 Jan 25;19:44. doi: 10.1186/s12888-019-2017-7 (PMC6347824; doi:10.1186/s12888-019-2017-7)
Supplement: Supplementary file 11 — Four patterns of suicide attempts within 12 months in the active contact and follow-up group. (DOCX 33 kb) [file 12888_2019_2017_MOESM11_ESM.docx]

**Table S10 Four patterns of suicide attempts within 12 months in the active contact and follow-up group**

|  | Pooled RR (95% CI) | |
| --- | --- | --- |
|  | Consensus[^7^](#_ENREF_7) | ITT[^7^](#_ENREF_7) |
| Medical record[^5^](#_ENREF_5) | 0.89 (95% CI = 0.75-1.05) n=6771 in 11 trials | 0.90 (95% CI = 0.76-1.07)  n=7561 |
| Self report^[5](#_ENREF_5)^ | 0.84 (95% CI = 0.74-0.96)* | 0.86 (95% CI = 0.73-1.02) |

Morthorst et al.^[5](#_ENREF_5)^ reported results from two different measures of repeat suicide attempts: medical records and patient self-reports.

Hatcher et al.^[7](#_ENREF_7)^ used the Zelen design and analyzed data using two different analysis sets. The analysis used two eligibility criteria. One was an intent-to-treat (ITT) analysis set: This analysis (n = 737 in intervention group and n = 737 in treatment as usual group) included participants who did not consent to receive intervention (n = 410) and those who did not consent to be followed up (380). The other analyses included only participants who consented to receive their intervention (n = 327) and those who consented to receive treatment as usual and to be followed up (n = 357).

*p = 0.0096.
